# Placebo effect after visual restitution training: no eye-tracking controlled perimetric improvement after visual border stimulation in late subacute and chronic visual field defects after stroke

**DOI:** 10.3389/fneur.2023.1114718

**Published:** 2023-06-29

**Authors:** Michael Christian Leitner, Anja-Maria Ladek, Florian Hutzler, Herbert Reitsamer, Stefan Hawelka

**Affiliations:** ^1^Salzburg University of Applied Sciences, Salzburg, Austria; ^2^Centre for Cognitive Neuroscience (CCNS), University of Salzburg, Salzburg, Austria; ^3^Department of Psychology, University of Salzburg, Salzburg, Austria; ^4^Research Program for Experimental Ophthalmology and Glaucoma Research, Department of Ophthalmology and Optometry, University Hospital of the Paracelsus Medical University Salzburg, Salzburg, Austria; ^5^Department of Ophthalmology and Optometry, SALK, University Hospital of the Paracelsus Medical University Salzburg, Salzburg, Austria

**Keywords:** stroke, visual field, rehabilitation, eye tracking, virtual reality, restitution training, neuroplasticity, visual cortex

## Abstract

**Introduction:**

A significant number of *Restitution Training* (RT) paradigms claim to ameliorate visual field loss after stroke by re-activating neuronal connections in the residual visual cortex due to repeated bright light-stimulation at the border of the blind and intact fields. However, the effectiveness of RT has been considered controversial both in science and clinical practice for years. The main points of the controversy are (1) the reliability of perimetric results which may be affected by compensatory eye movements and (2) heterogeneous samples consisting of patients with visual field defects and/or visuospatial neglect.

**Methods:**

By means of our newly developed and validated Virtual Reality goggles *Salzburg Visual Field Trainer* (SVFT) 16 stroke patients performed RT on a regular basis for 5  months. By means of our newly developed and validated *Eye Tracking Based Visual Field Analysis* (EFA), we conducted a first-time full eye-movement-controlled perimetric pre-post intervention study. Additionally, patients subjectively rated the size of their intact visual field.

**Results:**

Analysis showed that patients’ mean self-assessment of their *subjective* visual field size indicated statistically significant improvement while, in contrast, *objective* eye tracking controlled perimetric results revealed *no* statistically significant effect.

**Discussion:**

Bright-light detection RT at the blind-field border solely induced a placebo effect and did not lead to training-induced neuroplasticity in the visual cortex of the type needed to ameliorate the visual field size of stroke patients.

## Introduction

In 2019 approximately 12.2 million people sustained a stroke and reports indicate an estimated prevalence of 101 million cases worldwide (1). 20 to 50% of stroke patients and 12% of patients with Traumatic Brain Injury are diagnosed with hemianopia, that is, blindness in half of the visual field (2–4). Furthermore, around 29% of these patients are affected by so-called incomplete hemianopia [i.e., quadrantanopia (5)]. Patients have difficulties in reading, finding objects, navigating through traffic or face-to-face communication (4–6). These visual field defects (VFD) usually originate from lesions in *early* visual cortical areas–also called V1 or primary visual cortex–and the optic radiations (7). The most common reason for lesions after stroke in these parts of the brain is occlusion of the posterior cerebral circulation [e.g., (7, 8)].

An associated clinical disorder to VFD is (visuospatial) neglect. In contrast to VFD, neglect usually originates from lesions in *higher* cortical areas, located in parietal parts of the brain and due to arterial occlusion of the middle cerebral artery [e.g., (9, 10)]. Prevalence of neglect is approximately 30% after unilateral stroke (11) and a common comorbidity to VFD (12). While visual field defects are classified in the scientific literature as deficits in *perception*, visuospatial neglect is widely described as a deficit in *attention* [e.g., (13, 14)]. Another clinical disorder that is relevant in this context is “blindsight,” describing the phenomenon that some patients with VFD respond correctly to certain (emotionally salient) stimuli in their blind fields above chance level [e.g., (15, 16)]. However, the exact background for “blindsight” is not yet fully understood [e.g., (17)].

### Rehabilitation

*Visual Field Recovery*, *Vision Restoration Therapy* or simply *Restitution Training* (RT) are therapeutic concepts based on animal studies indicating that damaged neurons in the visual cortex can recover through repeated light stimulation (18, 19). Under the term RT exist different therapeutic concepts and methods, which range from motion discrimination of visual stimuli [e.g., (20)] to visual stimuli with different temporal and spatial frequency [e.g., (21)] and repeated visual stimulation along the area between the intact and defect visual field [e.g., (22)]. The present study focuses on the best-known therapeutic methodology of RT, namely repeated visual stimulation of the transition area between intact and defective visual fields, which is based on the “residual vision activation theory.” According to this influential concept, such reactivatable cortical areas are found, among others, along the “visual field border area” (23).

Proponents of RT argue that by stimulating the border area between the intact and damaged visual field with bright light impulses through the eyes - neurons in the corresponding (retinotopic) area of the visual cortex are reactivated - increasing the size of the intact visual field [e.g., (24, 25)]. Thus, changes after therapy with RT are perimetrically assessable [e.g., (22, 26)].^1^

Some studies report considerable training effects indicating training-induced neuroplasticity in the visual cortex [e.g., (22, 26–32)]. Bergsma et al. (22) observed in their explorative study perimetrically assessable reductions of a visual field defect to varying degrees in all their 12 patients with hemianopia after RT, which was performed for 1 h a day, 5 days a week and a period of 13 weeks at home. Marshall et al. (26) show an average improvement rate of 12.5% of perimetric stimulus detection in a longitudinal cohort analysis of 7 patients with VFD after conducting RT twice daily for 20 to 30 min, 6 days a week for 3 months at home. Mueller et al. (29) report in their clinical observational study with 302 patients that RT restored up to 17.2% of their patients’ formerly blind visual fields. Results are based on clinical standard perimetry after patients conducted RT daily for 1 h, 6 days a week for 6 months at home. Poggel et al. (30) included in their pre-post intervention study without a control group 9 patients with VFD who performed RT sessions of around 15–20 min for 3 months. The authors found slight but significant improvements in light detection performance. Matteo et al. (33) conclude in their review that “[...] *border rehabilitation seems to improve the detection of visual stimuli* [...]” (p. 1). Bergsma et al. (22) state that “*Visual restorative function training does not only lead to visual field enlargement* [...] *but it may also lead to subjective improvement of daily visual functioning* [...]” (p. 400).

Other studies, by contrast, did not find significant effects of RT [e.g., (34–37)]. Mödden et al. (36) found in their Randomized Controlled Trial of 45 stroke patients no perimetrically assessable visual field expansion after 15 patients performed RT in 15 single sessions for 30 min for over 3 weeks. Reinhard et al. (37) performed a pre-post intervention design with 17 patients with VFD who underwent RT 1 h a day, 6 days a week for 6 months. They conclude that “*in none of the patients* […] *an explicit homonymous change of the absolute field defects border* [was] *observed after training*” (p. 30). Raemaekers et al. (38) perimetrically identified increased visual fields after RT but found that corresponding fMRI data “[...] *could not account for the large increases in visual field size that were observed in some patients*” (p. 872). Frolov et al. (39) attest that there are *“remaining nagging questions as to the validity of* [published data] *and the clinical benefit*” (p. 40), and thus the effectiveness of RT remains unclear (40).

### Discrepancy

The first main reason for skepticism regarding the efficacy of RT is the imprecision of existing diagnostic instruments used in previous studies to assess the extent and potential amelioration of visual field loss. Automated static perimetry, like the Humphrey® Field Analyzer (HFA)–the gold standard in clinical visual field assessment–do not offer sufficient accuracy–due to the limited number of displayed stimuli positions. In addition, these devices do not have an automatically continuous and stringent eye fixation control to clearly exclude eye movements for compensation of visual field deficits. Although automatic static perimetry systems sometimes offer some sort of technical control mechanism (e.g., blind spot stimulation) or visual control of eye fixation for the examiner, there is no reliable control for every (small) eye movement, (short) fixation loss or quick saccadic search behavior. In clinical practice, these inaccuracies might be acceptable for the benefit of a quick diagnosis. However, in a scientific context where the central question is about improvements after a neuropsychological intervention in the range of a single-digit degree of visual field, such inaccuracies stemming from compensating eye movements lead to false impressions of a larger visual field. This is especially true if a therapy elicits high hopes in the patients and trains them to pay attention to peripheral stimuli. In this context, another significant technical issue is that the HFA uses the so-called SITA (Swedish interactive thresholding algorithm) to shorten the diagnosis in clinical routine. This means that based on the patient’s age and neighboring test points, this algorithm estimates - based on a database - the luminance threshold of every test point [e.g., (41)]. This represents a sensible method in everyday clinical practice, but a source of potential biases in scientific studies where the focus is on the most exact assessment of potential improvements after a neuropsychological intervention.

Goldmann Perimetry (GP) offers no remedy either as GP is characterized by low retest reliability, as even the inventor of the device himself - Hans Goldmann - states that “*[...] perimetry, and in particular kinetic perimetry, is an art. If one lets several young assistants examine the same patient* [...] *one will be astonished, even shocked about the difference in the results. It needs a long period of training until the results of two clinicians are comparable.* “[Goldmann quoted after (42), p. 3]. Even the application of alternative, non-standard diagnostic instruments such as “microperimetry” could not resolve the controversy. To illustrate, Marshall et al. (26) and Reinhard et al. (37) both used microperimetry to examine the effects of RT. While Marshall et al. (26) found “*modest but real expansions in visual fields*” (p. 1027), Reinhard et al. (37), in contrast, observed–“*in none of the patients*” - a “*change of the absolute field defect* [...] *after training*” (p. 30). Against this background, Frolov et al. (39) emphasized that “*the assessment of any potential visual restoration technique ultimately must rely on a reproducible and appropriate perimetric method.*” (p. 36). Summarized, the main problem is the lack of a precise and reliable perimetric method that adapts for patients’ compensatory eye movements, particularly rapid (unconscious) saccades.

The second main reason for skepticism regarding the efficacy of RT is the selection of patient samples and the empirical framework, especially in larger studies. For example, Mueller et al. (29) included 302 patients with lesions in post-chiasmatic and/or pre-chiasmatic pathways due to stroke, trauma, tumor, or anterior ischemic optic neuropathy. These inclusion criteria allowed for a wide range of comorbid medical conditions (e.g., neglect). Similarly, Romano et al. (31) included 161 patients with homonymous visual field defects after post-chiasmatic insult, but provided no further differentiation of affected brain regions. Smaller studies–such as from Poggel et al. (30) with 9 patients–also show heterogeneous patient samples. Eight patients were diagnosed with postchiasmatic lesions leading to homonymous hemianopia, but one patient had optic nerve damage after a tumor surgery leading to bilateral heteronymous loss of vision. Gall et al. (43) included 85 patients with significantly differing etiology, ranging from ischemic infarction, traumatic brain injury, hemorrhagic infarctions, encephalitis, anterior ischemic optic neuropathy and arteritic optic nerve infarction. Additionally, 69 of 85 patients paid privately for participating in the training leading to authors’ and patients’ competing and personal interests. Summarized, a clear investigation of the efficacy of RT was obstructed by heterogeneous patient samples. As a result, comorbid disorders (e.g., neglect, prechiasmatic lesions) have confounded–in a considerable number of previous studies - an accurate analysis of the therapy’s potential effect on neuronal regeneration in early cortical areas.

### Originality

The present study is the first of its kind to quantify the therapeutic effect of RT utilizing a specially developed and validated eye-tracking-based perimetric methodology that fully corrects for compensatory eye movements in real time (44). Also, it is the first study to include a highly selective sample of patients with visual field defects originating from lesions in early cortical areas, who conducted RT using VR goggles, which we specifically developed for this study (45).

## Methods

To make accurate conclusions about the effects of RT and to avoid potential inadequacies in patient selection, rehabilitation, and diagnostics, we (1) limited inclusion criteria for study participants, (2) used our (beforehand) newly developed and validated highly accurate eye-tracking assisted perimetric instrument (44) for patients’ visual field analysis, and (3) utilized our also newly developed, validated, highly reliable and easy to use virtual reality rehabilitation instrument enabling patients to perform RT at home (45).

### Inclusion criteria

(1) Acquired post-chiasmatic anopia (H53.4; ICD-10), (2) Lesioning of the occipital lobe, specifically primary visual cortex or optic radiations due to, e.g., stroke from thrombosis of the posterior cerebral artery (I63.33; ICD-10) or similar etiology [e.g., (7, 46)] (3) Perimetrically diagnosed “Chronic visual field defect” for a minimum of 3 months since clinical incident (7, 39, 47) (4) Older than 18 years.

### Exclusion criteria

(1) Patients with diagnosed cognitive deficits such as disorder of awareness, speech production, anosognosia, severe attentional problems, problems understanding and following instructions (e.g., R41.x, R44.x, F06.7; ICD-10) after significant temporal and/or parietal lobe damage (7) (2) Patients with unilateral visuospatial neglect (R29.5; ICD-10)

### Selection procedure

Patients were recruited via our partnering rehabilitation institution (Rehabilitation Center Grossgmain), training partner (Club Mobil) and with the help of press reports to the public. Information regarding our study was also disseminated by the Paris Lodron University Salzburg and the University Clinic Salzburg. Eligible patients were first interviewed in person or via telephone, comprehensive individual medical histories were obtained, and inclusion and exclusion criteria were assessed accordingly. These anamnestic data and neuropsychological examinations performed during previous hospitalization and rehabilitation were utilized to exclude confounding comorbidities and to ensure reliability and validity of visual field assessment and the self-performed execution of RT.

### Patient sample

A total of 16 patients were included. This number is greater than in comparable studies who found significant improvements of stroke patients’ damaged visual field after RT [e.g., (22, 26, 30)]. Fourteen patients sustained cerebral infarction due to thrombosis of the posterior cerebral artery (I63.33; ICD-10). One patient additionally had middle cerebral artery ischemia (I63.33; ICD-10 and I63.51, ICD-10) and one patient was diagnosed with basal ganglion hemorrhage (I61.3, ICD-10).

### Privacy and ethical considerations

The study was approved by the ethics committee of the University of Salzburg (Reference No. 39/2018) and guided by the fundamental principles of respect for the individual, the right to self-determination and informed decisions, i.e., informed consent, as noted in the Declaration of Helsinki. The study was registered in the ICMJE-approved registry *German Clinical Trials Register* (DRKS00025205).

### Experimental design overview

The study qualifies as a clinical pre-post intervention study with 16 patients diagnosed with quadrantanopia or hemianopia after cortical lesions in early visual areas (see Table 1 for details). After an initial assessment - consisting of visual field diagnostics with a Humphrey Field Analyzer (HFA) and Goldmann Perimetry (GP)–the patients’ visual border area between intact and defect visual field was precisely assessed with the Eye Tracking Based Visual Field Analysis [EFA; (44)]. We focused on the inner 10 to 15° for three main reasons. (1) Central foveal areas are represented in the early visual cortex (V1) with a significantly larger area of cortical surface than more peripheral, outer areas (48). Following the logic of RT, the higher the number of neurons, the higher the probability of new connections between them. (2) By limiting diagnostic effort on these inner areas, we kept visual field assessments short and prevented erroneous behavioral responses due to cognitive overload, stress or fatigue. (3) Previous studies showed that improvements after RT ranged around 5 to 10 degrees of visual angle. Improvements of this size would be especially desirable in central areas of the visual field.

**Table 1:** Demographic and clinical data of the included stroke patients.

	Code	Age	Sex	Time since stroke (in months)	ICD-10	Affected hemisphere	Visual field loss
	01	24	f	14	I63.33	Right	Upper Quadrantanopia
	02	76	m	202	I63.33	Left	Hemianopia
	03	78	f	15	I63.33	Right	Lower Quadrantanopia
	04	64	m	39	I63.33	Left	Hemianopia
	05	88	m	36	I63.33	Right	Hemianopia*
	06	78	m	6	I63.33	Right	Upper Quadrantanopia
	08	54	m	44	I63.33	Right	Hemianopia
	10	36	m	3	I63.33	Left	Lower Quadrantanopia
	11	31	m	22	I63.33	Right	Hemianopia
	12	54	m	13	I63.33	Right	Hemianopia
	13	53	m	15	I63.33	Left	Upper Quadrantanopia
	14	53	m	18	I61.3	Right	Hemianopia
	17	57	f	6	I63.33	Left	Hemianopia
	19	57	m	19	I63.33, I63.51	Right	Hemianopia
	20	39	f	3	I63.33	Left	Hemianopia
	22	35	m	4	I63.33	Left	Hemianopia
Mean		55		29			
SD		18		46			
N	16		*f* (4)/m (12)		I63.33 (14)/I63.33, I63.51 (1)/I61.3 (1)	Right (9)/left (7)	Quadrantanopia (5)/Hemianopia (11)

ICD-10: I63.33 posterior cerebral artery infarction, I63.51 middle cerebral artery infarction, I61.3: basal ganglion hemorrhage/*Due to ptosis, diagnosis was performed with the left eye only.

Results from the EFA provided the basis for individual rehabilitation configuration of the Salzburg Visual Field Trainer [SVFT; (45)] for every patient. Patients were extensively educated on the usage of the SVFT and the functionality of RT and instructed to exercise for 5 months, 6 times a week, 2 times a day for 30 min (SVFT was configured to automatically end training after 30 min). Training regime and sample size was based on previous studies reporting amelioration after RT [e.g., (22, 26, 29, 49)]. After around 2 months, the patients’ visual field was perimetrically reassessed using the EFA in order to adapt the training stimuli in the SVFT to potential changes in visual border characteristics.

### Experimental procedure

The aim of the experiment was twofold: First, we investigated whether the visual field of stroke patients *objectively* increases after rehabilitation with RT. Second, a single question was used to determine whether the patients would experience/perceive a *subjective* increase of their intact visual field. The question was: “How do you rate the current status of your visual field?.” The potential answer ranged from “very bad” to “very good” and was provided on a horizontally aligned Visual Analog Scale (VAS). The methodology of VAS is a validated psychometric approach and widely used for subjective measurement of emotions, sensations, and other subjective feelings [e.g., (50)].

**Figure 1:**
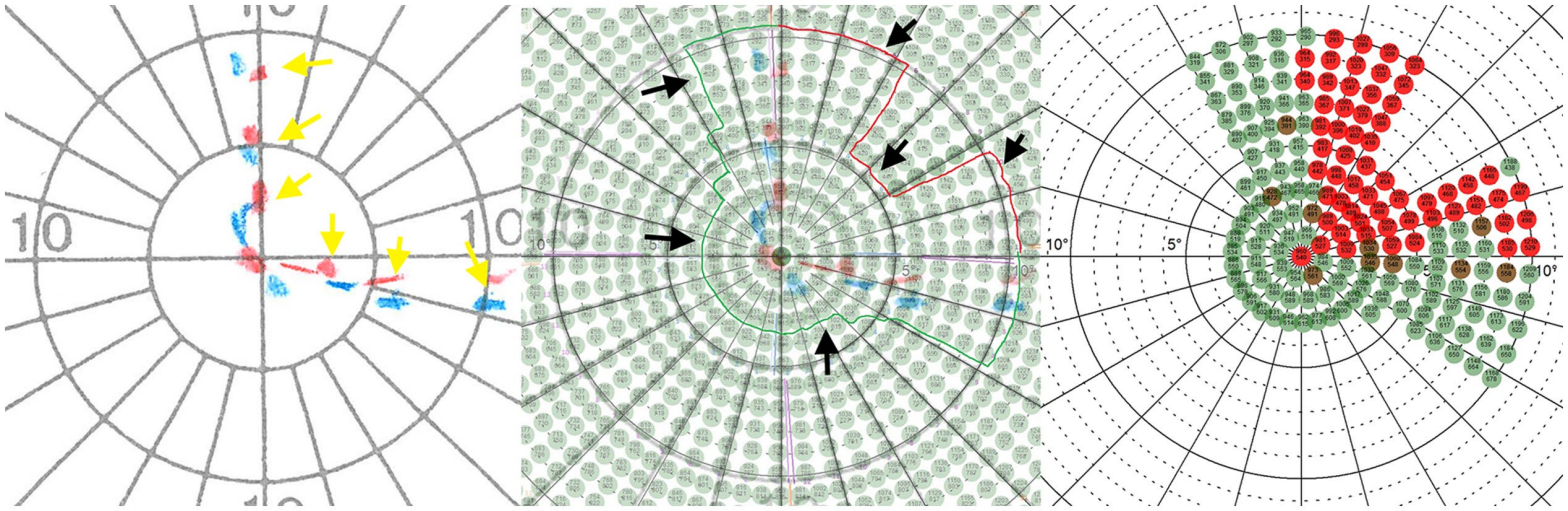
Left: The red (Marker properties: 4, e, III) and blue (Marker properties: 3, e, III) dotted lines (marked with yellow arrows) show an exemplary patient’s visual border area resulting from assessment with Goldmann perimetry (GP). Middle: The GP plot was superimposed with the coordinate system of the Eye Tracking Based Visual Field Analysis (EFA) to define a surrounding visual area (marked by black arrows) that was reexamined at high resolution and with real-time gaze contingent stimulus adaptation of the EFA. Right: Perimetric EFA plot which was used for the pre-post analysis. Alt Text: Using the results from Goldmann perimetry, the location of the transition area between intact and defect visual field was defined and reassessed with high resolution and real-time gaze contingent stimulus adaptation using the Eye Tracking Based Visual Field Analysis (EFA).

In an initial assessment session, the visual field of the included patients were measured by a trained ophthalmologist using conventional methods [Humphrey Field Analyzer (HFA) and Goldmann Perimetry (GP)]. This gave us an initial insight into the perimetric status of the patients and enabled configuration for the next diagnostic step. In this second assessment session and based on the results from GP, the inner visual field area (10–15 degrees of visual angle) of the patients’ respective border area were assessed in high resolution (1 stimulus = 1 angular degree) using our *Eye Tracking Based Visual Field Analysis* [EFA; (44)]. Figure 1 shows exemplary patient’s data and the logic of translation from GP to EFA.

The EFA is a computerized visual field test based on the principles of classical automated static perimetry. The special feature of the EFA, however, is the continuous eye fixation control, which (1) checks throughout whether the patient is fixating centrally and (2) compensates any deviation in real time by adapting the variance to the position of the currently presented test stimulus. Specific care was taken to ensure that the individual partial assessment with the EFA did not last longer than 15 min. Depending on the size of the visual subareas to be examined, breaks were scheduled to avoid generating assessment errors due to overexertion. In addition to automated real-time adjustment of test stimuli depending on patient eye movements, the EFA also controls the timing of responses to the presented stimuli. The presentation of the stimuli is varied using a randomized time interval. For example, if patients systematically press the response button - even though no stimulus was presented - the first three times the patient is instructed with a text not to do so. In addition, this behavior is counted automatically, even if no warning is displayed on the screen after the third time. For detailed information on the EFA see Leitner et al. (44).

**Figure 2:**
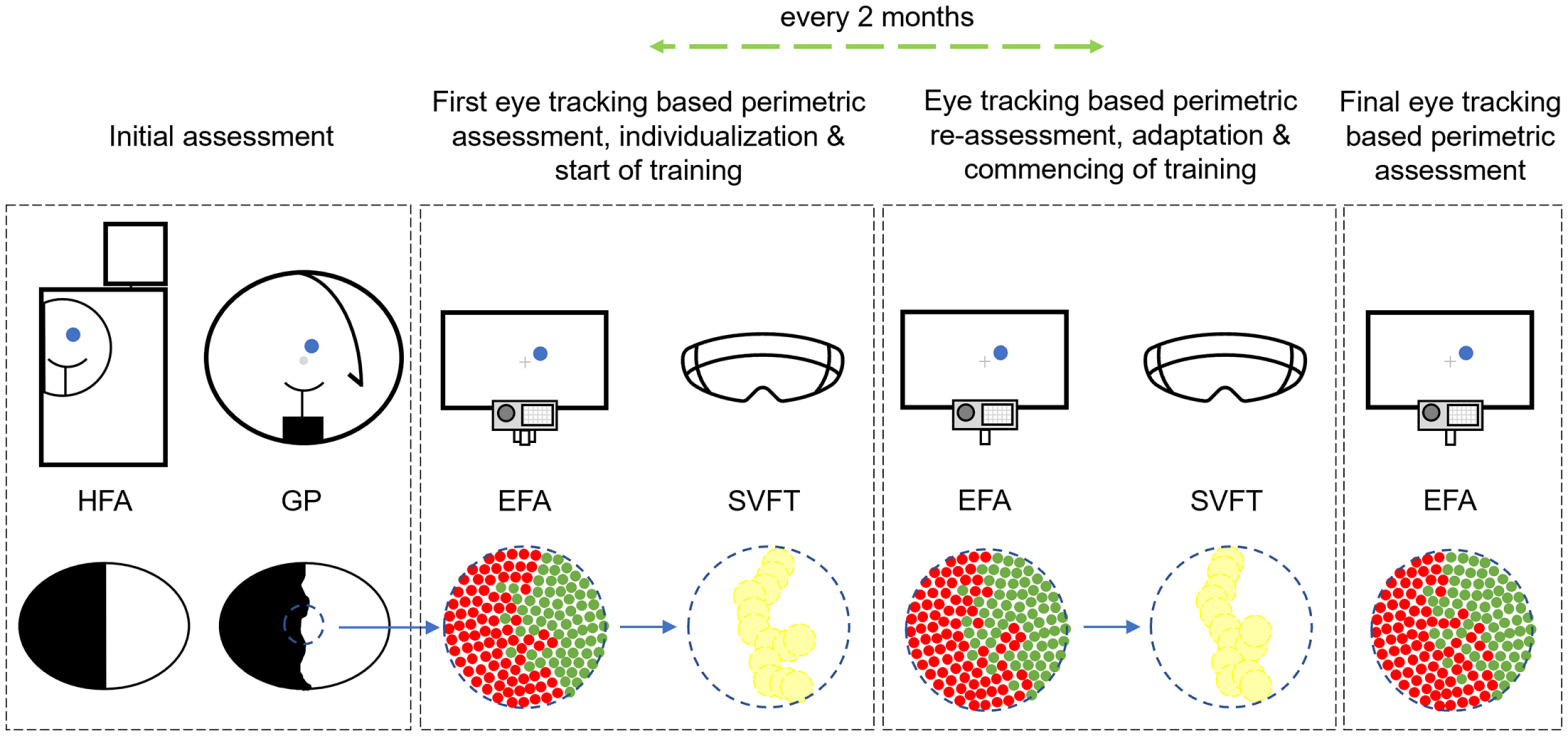
Experimental sequence of perimetric assessment with Humphrey Field Analyzer (HFA), Goldmann Perimetry (GP) and Eye Tracking Based Visual Field Analysis (EFA) with subsequent individual implementation into the Salzburg Visual Field Trainer (SVFT) with which the patients performed Restitution Training. Alt Text: The study’s procedure showing the different steps of perimetric assessment to assess the patients’ visual field before and after Restitution Training and to configure the Virtual Reality goggles “Salzburg Visual Field Trainer” depending on patients’ individual visual field loss.

Besides usage for pre-post assessment and investigation for potential changes after rehabilitation with RT, perimetric EFA results were also used for the individual placement of training stimuli (1 stimulus = 3 angular degrees) on the patients’ visual border area in the *Salzburg Visual Field Trainer* (SVFT). The SVFT is a virtual reality device–based on Google cardboard systems–for which we developed training software based on the principle of RT. For detailed information on the SVFT see Leitner et al. (45). Once the SVFT training goggles were configured to each patient’s individual border area location and extent, patients were given detailed instructions and education about the training regime, the logic behind RT, and the SVFT itself. We paid special attention to the fact that the patient should maintain a continuous fixation on the central fixation cross during the training. We repeatedly drew the patient’s attention to the fact that this must be always complied with. Every 2 months, an intermediate perimetric assessment was performed and–if necessary–the localization of the training stimuli in the SVFT were adjusted accordingly in case shifts in the visual border area were found. After completion of the intervention a final visual field assessment was performed. The described intervention procedure was not changed throughout the duration of the experiment. However, due to the COVID-19 pandemic some interim assessment dates had to be postponed. Consequently, the time between some intermediate assessments were shorter or longer than 2 months (also see the Results section and the Supplementary material). Figure 2 shows a summary of the experimental procedure.

### Perimetric data analysis

Perimetric results from the EFA were classified as follows (based on (44)): (1) Perceived stimuli, (2) partially perceived stimuli and (3) unperceived stimuli. If the patient reacted to a presented stimulus, it was documented as perceived. If a stimulus was not perceived it was repeated after one randomized intermediate stimulus (which was excluded from analysis). If the stimulus was not perceived the first time but perceived at repetition, it was classified as a partially perceived stimulus. If the stimulus was not perceived both times, the stimulus was defined as unperceived. EFA plots illustrate perceived stimuli in green, partially perceived stimuli in brown and unperceived stimuli in red. For data analysis, perceived stimuli were calculated with 1 point, partially perceived stimuli with 0.5 points, and unperceived stimuli with 0 points.

In every first EFA assessment, a tight clustering of a generous number of stimuli was used to assess the exact border between defect and intact visual field as approximated from data of HFA and GP. Thereby, in later sessions, the number of test stimuli could be reduced in areas known to be clearly intact or defect. In order to ensure comparability between assessment dates, only test stimuli that were utilized in *all* assessment sessions were included for data analysis. This ensured no under-or overestimation of potential visual field changes.

The closer a visual field defect lies foveally, the more distressing for the affected patient. Especially in central areas where visual acuity is high, even a few angular degrees of improvement would be crucial for the patient. For this reason, we additionally calculated patients’ perimetric results specifically within 0° to 5° by weighting the decrease in acuity into analysis. This means that the closer a test stimulus was displayed foveally, the higher it was weighted in the analyzes [e.g., (51)]. Accordingly, test stimuli from 0° to 5° were weighted with the following values (in brackets): 0° (1), 1° (0.697), 2° (0.535), 3° (0.434), 4° (0.365), 5° (0.315).

### Subjective data analysis

Each end of the VAS scale regarding the subjective perception of the patients’ subjective status of his/her own visual field loss was defined by contrasting terms ranging from “very bad” (0%) to “very good” (100%). The position of the marking on the VAS made by the patient was measured, converted into the corresponding percentage value, and statistically analyzed.

## Results

### Number of assessments, training, and diagnostics reliability

On average, we perimetrically examined all patients for 3.1 times (SD = 0.6), with a mean of around 154 days (SD = 66.9) between first and last assessment date. Analysis of rehabilitation documentation from the SVFT records show that patients performed on average 1.4 (SD = 0.4) training sessions per day (also see Table 2).

**Table 2:** Patients’ training statistics with the SVFT.

	Total number of perimetric assessments	Days between first and last perimetric assessment	Total number of training sessions	Mean training sessions per day
Mean	3.1	153.9	204.4	1.4
Median	3.0	140.0	220.0	1.6
SD	0.6	66.9	74.6	0.4
Min	2.0	56.0	83.0	0.4
Max	4.0	364.0	346.0	1.8

Perimetric control values of the EFA did not show any conspicuous accumulations regarding “trigger happiness” in any patient. The number of mean false responses was 0.9 (SD = 1.1; MIN = 0; MAX = 5) in all perimetric assessment sessions.

### Subjective assessment

**Figure 3:**
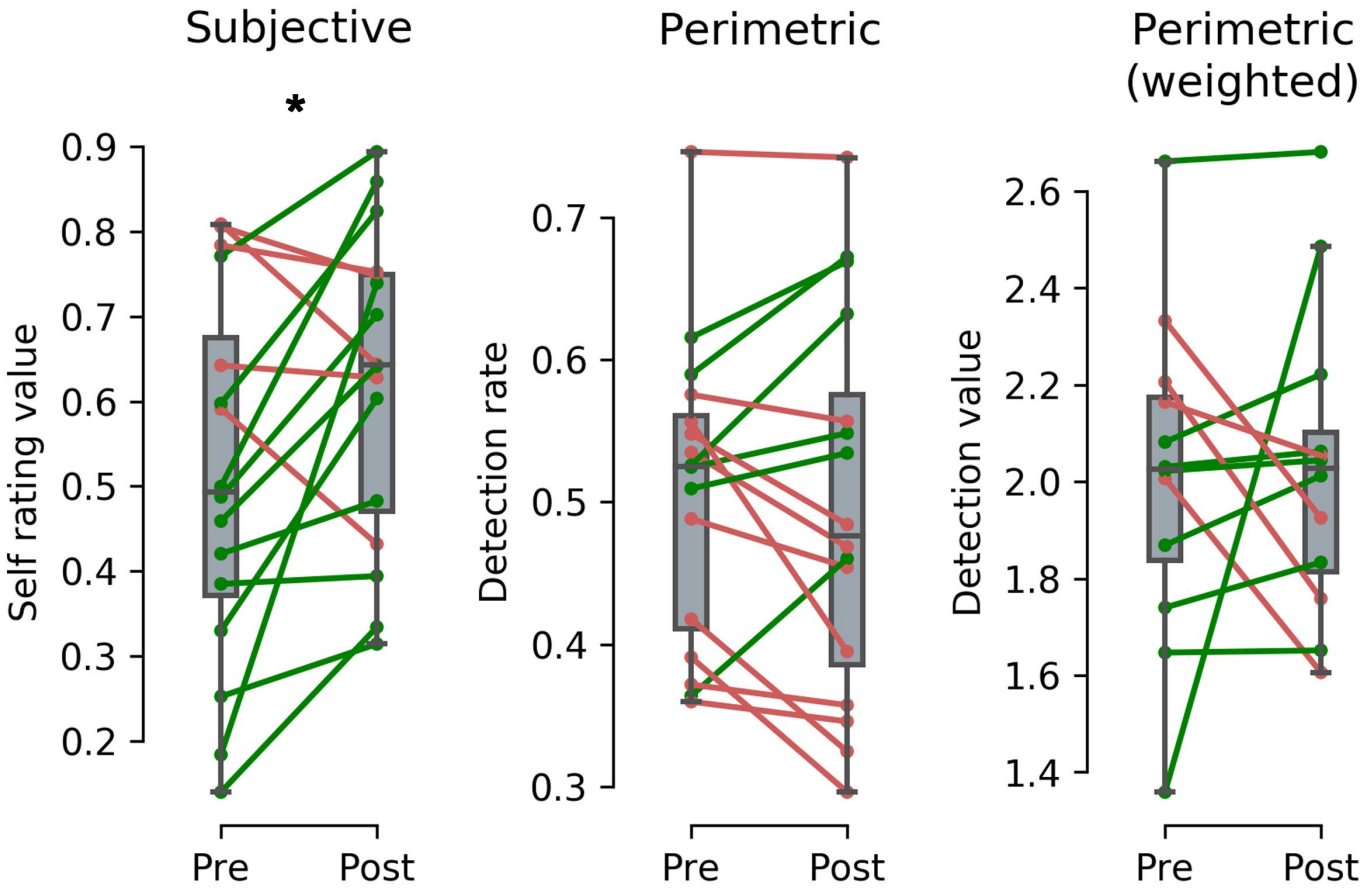
Difference between pre-post Restitution Training (RT) results after a mean training duration of around 154  days and 204.4 training sessions from subjective (left panel) and perimetric (middle panel) assessment of 16 stroke patients and acuity weighted perimetric assessment (right panel) of 12 stroke patients. Subjective results show a perceived improvement of intact visual field size of 11.5% - a statistically significant effect. In contrast, results from perimetric and acuity weighted perimetric results show *no* statistically significant difference with a mean change of −1.1% in stimulus detection rate and 0.02 in calculated stimulus detection value, indicating a placebo effect in RT. (* = *p* < 0.05). Alt Text: Patients’ subjective, perimetric and weighted perimetric results before and after Restitution Training illustrated as paired plots.

Evaluation of subjective self-assessment regarding the size of intact visual field shows that patients reported a mean percentile improvement of 11.5%–changing from 51.0% (SD = 21.2) before RT to 62.4% (SD = 17.9) after completing RT (see left panel of Figure 3). Kolmogorov–Smirnov test (KS) indicates normal distribution and paired samples t-test shows a statistically significant difference and a medium to large effect size between first and last assessment date; t(15) = −2.356, 95% CI [−0.215, −0.011], *p* = 0.032, r = 0.52.

### Perimetric assessment

Analysis of patients’ visual field tests shows that mean stimulus detection rate before RT was 50.7% (SD = 10.6) and after RT 49.6% (SD = 13.1; see middle panel of Figure 3). KS indicates normal distribution and paired samples t-tests show *no* statistically significant differences between perimetric pre-post assessment; *t*(15) = 0.590, 95% CI [−0.288, 0.051], *p* = 0.564 (For details on the individual patients see the Supplementary material).

### Eccentricity weighted perimetric assessment

Note that only patients with defects in the inner 5° qualified for the following analysis (*N* = 12): Eccentricity weighted analysis of potential changes near the macula before and after RT shows that the calculated mean stimulus detection value before RT was 2.01 (SD = 0.34) and after RT 2.03 (SD = 0.32) (see right panel of Figure 3). KS indicates normal distribution and paired samples t-tests show *no* statistically significant differences between perimetric pre-post assessment; *t*(11) = −0.152, 95% CI [−0.280, 0.243], *p* = 0.882.

## Discussion

The present study is the first of its kind to examine the potential effects of *Visual Field Recovery*, *Vision Restoration Therapy* or simply *Restitution Training* (RT) (and similar methodologies) by utilizing a novel, clinically validated eye tracking based methodology (Eye Tracking Based Visual Field Analysis; *EFA*) in a highly controlled perimetric manner (44). Furthermore, by also developing and validating beforehand a portable and flexible virtual reality device (Salzburg Visual Field Trainer; *SVFT*), we ensured that patients could easily, comfortably, and accurately perform RT at home (45). Sixteen patients with visual field loss due to lesions in the primary visual cortex and/or optic radiations after stroke performed RT with the SVFT on a regular basis for 5 months and were perimetrically assessed with the EFA before, during and after rehabilitation. On an individual basis, 10 patients showed slightly worse perimetric results and 6 patients slightly better perimetric results after training. On the group level, this does *not* result in a statistically significant, systematic perimetric change after intervention with RT. Thus, our findings indicate that RT is an *ineffective* therapeutic approach to systematically and perimetrically assessable increase the visual field of stroke patients with lesions to the primary visual cortex and/or optic radiations. We conclude that this is because RT does not restore neuronal connections in visual cortical areas, as proposed by proponents of RT. Rather, our findings on the patients’ *subjective* evaluation of their visual field improvement after RT indicate that RT is based on a psychological *placebo effect*. Summarizing, our findings allow three conclusions on RT.

1. Patients *overestimate* training effects of RT when they subjectively evaluate the size of their intact visual field on a rating scale
2. Perimetric results from the EFA show that RT does *not* improve or restore damaged visual field areas after lesions in the primary visual cortex or optic radiations
3. When results are weighted for eccentricity - that is, depending on test stimulus distance from the macula, representing the natural parabolical decrease of acuity - *no* improvement in visual field size within 0° to 5° is evident in perimetric results from the EFA

Clinical evidence to date on the effect of RT on cerebral neuroplasticity using neuroimaging methods is limited. In a review from 2016, Matteo and colleagues identified two clinical studies related to the assessment of cortical functions after RT: Julkunen et al. (52) investigated five stroke patients with visual field defects and found indications of an improvement in three patients’ visual evoked potentials after RT. In 2006, the same workgroup investigated one stroke patient with visual field defect and found a previously absent P100 component and - by utilizing Positron Emission Tomography (PET)–an increase in regional cerebral blood flow in occipital areas after RT (53). In this context, it seems noteworthy that despite the apparent limited number of neuroimaging studies on RT around that time, a much larger number of studies argued–as described in the Introduction–in favor of the positive effect of RT on “training-induced neuroplasticity” and related perimetrically detectable improvements in the past.

Even now in 2023, there is only a limited number of clinical neuroimaging studies on the neurological effects of RT. For example, Ajina et al. (54) included seven stroke patients with chronic visual field defects who performed RT daily (~25 min) for 3 to 6 months at home. The authors found “[…] *an increased neural response to moving stimuli in the blind visual field in motion area V5/hMT*” (p. 5994) and “*using a region-of-interest approach* […] *a significant effect on the blood oxygenation level-dependent signal compared with baseline*” (p. 5994). Contradicting findings, on the other hand, come from Barbot et al. (27), who found in 11 stroke patients with visual field defects after RT that “[…] *blind-field locations with the greatest HVF* [Humphrey Visual Field] *recovery did not exhibit further increases in visually-evoked BOLD responses post-training*” (p. 12). Similarly, Raemaekers et al. (38) found in their fMRI study on eight patients with postchiasmatic visual field defects perimetrical improvements between 1 and 7 degrees of visual angle after RT. However, their fMRI data revealed that “[…] *the retinotopic maps strongly matched perimetry measurements before training*” (p. 872).

In the presence of this small number of clinical neuroimaging studies to date, previous argumentation of proponents of RT and our first-time eye tracking based perimetric findings in the present study, we argue that following the postulated mode of action of RT, it is unlikely that RT leads to perimetrically assessable “training-induced neuroplasticity” in the primary visual cortex or the optic radiations. Consequently, a practical benefit of RT for the everyday life of patients must be seriously doubted. At the same time, it is surprising to see that a considerable number of newer studies, while relying on modern neuroimaging methods, still use–in scientific terms–rather “inaccurate” equipment such as the Humphrey Field Analyzer to assess–if at all minor–perimetric effects of RT [e.g., (27, 54)]. For a comparison on perimetric accuracy between Humphrey Field Analyzer (HFA), Goldmann Perimetry (GP), and Eye Tracking Based Visual Field Analysis (EFA) also see Leitner et al. (44).

Following the concept of RT, especially the visual transitional area between intact and defect visual field should be highly receptive for neuroplastic improvement due to retinotopy. This is because transition areas near the macula–which we stimulated in our study–are cerebrally represented with a higher number of intact neurons neighbored by (partially) damaged neurons than in peripheral areas. Additionally, especially defect neurons in macular areas on lesion borders should be more easily stimulated to reconnect, than neurons with scarcer input from peripheral retina located in the center of cortical lesion. Also, the “dual blood supply”–from the posterior cerebral artery and the middle cerebral artery–of the posterior occipital lobe play a significant role in both sparing and rehabilitation after stroke of the central 2° to 10° of the visual field (7).

While the neurological background of visual field defects due to lesions in the primary visual cortex and optic radiations is well understood, related visual disorders such as neglect and cortical blindness still raise questions. Since lesions do not follow cerebral-topographical borders, for example, the posterior cerebral artery rarely affects purely visual perception, the distinction between visual field defect, neglect and other associated disorders is often not clearly apparent. We therefore argue that a possible explanation for the partly divergent findings in previous studies on RT should be considered against this background. This hypothesis is also based on our recent findings on RT and neglect (55). We argue that RT could have a potential positive therapeutic effect for lesions in higher cortical areas, such as in parietal areas impairing attention. These improvements can be explained by training induced focus on specific areas of visual perception. However, we argue that the neurological background of these improvements are not necessarily training-induced reconnections between neurons but rather a neuropsychological phenomenon based on a strict training and repetition scheme improving attention and awareness - similar to the concept of *Compensation Training*. In this context, recent results by Halbertsma et al. (56) are of interest, finding in 20 patients with chronic hemianopia that “[…] *the functional connectivity strength between the anterior Precuneus and the Occipital Pole Network was positively related to the attention modulated improvement* [by RT]” (p. 1). Similarly, Lu et al. (57) found in their study on seven patients with post-chiasmatic damage and RT of 5 weeks significantly improved contrast sensitivity after assessment with HFA and enhanced functional connectivity of attentional brain regions. If findings like these can be confirmed in future RCT designs and larger samples, it will then be a central task to quantify the significance of improvement for the practical daily life of patients, as emphasized by Kerkhoff et al. (4).

We decided against conducting a larger “Quality of Life”-like questionnaire for perceptual and visual field disorders. The main reason is that these questions are not the focus of our present study as we did not seek to find answers regarding general and specific experiences in stroke patients’ everyday life dealing with visual field defects. Rather, the aim of this study was to test the hypothesis that neuropsychological intervention with RT ameliorates a damaged visual field. The answer to this question consists of a (1) *physiological* component, which was *objectively* assessed via perimetric measurement, and (2) a *psychological* component, which was *subjectively* assessed via a simple question: “How do you rate the current status of your visual field?” Interestingly, the result from this subjective perception of the patients–a significant improvement–coincides with other studies, which–in contrast to our study–also reported perimetric improvements after RT [e.g., (22, 58)].

One might argue that our failure to find affirmative evidence in favor of RT is due the application of RT in VR goggles (whereas those studies, which reported such evidence were conducted with standard monitors). We counter that VR provides the optimal framework for neuropsychological rehabilitation as more and more recent studies indicate the advantages and validity of neuropsychological interventions in a virtual reality-based environment [e.g., (45, 59–61)]. There are several reasons for the advantages of neuropsychological rehabilitation of visual perception and attention issues in VR: (1) Following the logic of RT, the brightness of the training stimuli represents a determinant measure for neuronal stimulation and thus therapeutic success. The stimuli presented in the SVFT have a brightness of 1,000 cd/m^2^. On the other hand, PC monitors utilized for RT have an average brightness of around 400 cd/m^2^. (2) Training can be conducted highly comfortably and convenient as no chin and head rest is required. As the distance between the eyes and the presented stimuli is always the same, head movements do not affect the crucial aspect of exact stimulus presentation across the visual border area (as in contrast to PC based RT systems). (3) The immersive design of the VR goggles enables ruling out external factors like light conditions or other visual distractions in the surrounding environment, potentially confounding the therapeutic effect. From a technological perspective, our proof-of-concept and validation study with 40 participants indicated beforehand that the RT program of the SVFT has a sensitivity of 0.980 (SD = 0.038) and a specificity of.992 (SD = 0.016). From a usability perspective our study showed that the VR system is comfortable to wear and easy to use (45).

Since there was no eye-tracker installed in the SVFT, we could not document the eye movement behavior of the patients during RT. Thus, we extensively informed the patients before the start of the training that only a reliable and continuous central fixation during RT can potentially yield an improvement. Furthermore, during each interim assessment - about every 8 weeks - the importance of central fixation was re-emphasized. In addition, we kept in touch with the patients by telephone to be able to help them quickly with any other problems that might arise during RT. Because the patients were thoroughly educated on the functionality of RT and had an honest intrinsic motivation to perform the therapy as correctly as possible, we assume a high level of compliance.

The definition of a “chronic visual field defect” is not consistent in the scientific literature ranging from 3 [e.g., (47)] to 6 months [e.g., (26)] and 12 months [e.g., (37)]. Based on recent work from Frolov et al. (39) or Goodwin (7) and older work from Zhang et al. (47) we decided to include patients who suffered from stroke at least 3 months in the past. Zhang et al. (47) state in their influential article on homonymous hemianopia that “*spontaneous improvement of homonymous hemianopia is seen in at least 50% of patients first seen within 1 month of injury. In most cases, the improvement occurs within the first 3 months from injury*” (p. 901). Because our patient sample is composed of late subacute and chronic visual field loss, we cannot draw conclusions about possible improvements with RT in earlier phases after stroke. There are some indications that earlier interventions (acute and early subacute phase) may lead to greater success in the restitution of visual field capacities [e.g., (62)].

Since the perimetric results from the EFA do not suggest efficacy of RT, we decided against conducting a control group. This is a decision of ethical nature. We argue that it is ethically not justifiable to misspend therapeutic time of stroke patients with obviously inefficient forms of rehabilitation. We also consider it as unethical to advise stroke patients against other, potentially effective forms of therapies while waiting in a control group or to perform pseudo-interventions for months, when previous, well-founded research has shown the ineffectiveness of an intervention. Although the placebo effect found in our study has thus not been validated with a control group, the state of current research on RT supports our conclusion on this psychological effect. In the past, numerous studies have also found–besides perimetric improvements–a subjective improvement based on interviews or questionnaires [e.g., (22, 28, 29)]. In this context, we are not aware of a single study that, when surveyed in the course of the study design, did not find a positive effect of RT on subjective patients’ perception on their perceived severity of visual field loss. Consequently, we argue that our study was able to reproduce the results of other studies, but puts these findings in the context of a placebo effect due to our first-time investigation with the EFA and its unprecedented perimetric reliability and accuracy. We would like to emphasize that we do not consider the placebo effect in the context of rehabilitation of visual field defects as “negative” for the patient. The placebo effect is a very well studied phenomenon and powerful tool that works even when patients know that the effect is based on a placebo [“open-label placebos”; e.g., (63, 64)]. Against this background, a placebo effect after VRT that leads to a subjectively improved visual field can even be considered positive from a clinical-psychological point of view. From an economic point of view, however, the question arises as to whether this effect can be achieved more effectively and in a less time-consuming manner than through an expensive and complex hardware/software system. This may warrant further research. Up to now, stroke research on visual field loss has focused primarily on physiological issues. Our study shows, however, that psychological effects may have a greater impact on the therapeutic success of affected patients than previously assumed.

Having said this, we lay emphasis on the fact that the finding of the current study–a null effect of visual restitution training - only applies to this very specific sort of RT. Our findings do in no way apply or touch other forms of interventions for visual field loss. There are promising approaches toward ameliorating visual field defects [see (65, 66) for a recent review]. A recent study, for example, evinced positive outcomes of training a new preferred retinal location (67). Others reported positive results with regard to training with concurrent brain stimulation (Transcranial Random Noise Stimulation; tRNS) leading to improved spatial attention and stimulus detection performance [e.g., (68–71)]. Although it is possible that a stringent eye fixation control would qualify the findings of these studies, their methodology seems promising with regard to alleviating the burden of cortical blindness.

## Conclusion

With our newly developed and validated instrument EFA we find no perimetrically assessable ameliorating effect of *Restitution Training* (RT) on the visual field of stroke patients’ due to lesions in the primary visual cortex and/or optic radiations in the late subacute (3–6 months) and chronic phase (> 6 months). Thus, RT seems to have no training-induced effect on cerebral neuroplasticity in these cortical regions. However, we find a statistically significant improvement in the patients’ *subjective* assessment regarding the size of their intact visual field. Consequently, we argue that the therapeutic impact of RT seems to be a psychological placebo effect, which gives patients the feeling that their intact visual field is larger than it actually is.

## Data availability statement

The original contributions presented in the study are included in the article/Supplementary material, further inquiries can be directed to the corresponding author.

## Ethics statement

The studies involving human participants were reviewed and approved by the Ethics commitee of the University of Salzburg (Ref.-No. 39/2018). The patients/participants provided their written informed consent to participate in this study.

## Author contributions

ML, FH, and SH: conception and design and writing publication. ML and A-ML: data acquisition. ML, A-ML, FH, HR, and SH: analysis and interpretation of data and critical revision of publication. ML and SH: supervision. FH and HR: resources. A-ML and SH: technical administrative and support. All authors contributed to the article and approved the submitted version.

## Funding

The following study is based on the funded project *Advanced Perimetry for the Evaluation of Neuroplasticity in the Visual Cortex* by the Austrian Science Fund (FWF; Reference No. P31299). The funding source had no role in the design of the study, the analysis and interpretation of the data or the writing of, nor the decision to publish the manuscript. Authors were not paid by a pharmaceutical company or other agency to write this article.

## Conflict of interest

The authors declare that the research was conducted in the absence of any commercial or financial relationships that could be construed as a potential conflict of interest.

## Publisher’s note

All claims expressed in this article are solely those of the authors and do not necessarily represent those of their affiliated organizations, or those of the publisher, the editors and the reviewers. Any product that may be evaluated in this article, or claim that may be made by its manufacturer, is not guaranteed or endorsed by the publisher.

## Footnotes

^1^In this context, there are numerous studies also dealing with the rehabilitation of neglect or “blindsight.” However, these are not the subject of the present study and are therefore not discussed.
