# Supplementary material for: Placebo effect after visual restitution training: no eye-tracking controlled perimetric improvement after visual border stimulation in late subacute and chronic visual field defects after stroke
Source: Front Neurol. 2023 Jun 29;14:1114718. doi: 10.3389/fneur.2023.1114718 (PMC10339290; doi:10.3389/fneur.2023.1114718)
Supplement: Supplementary file 1 [file Data_Sheet_1.PDF]

CODE: 01 / AGE: 24 / SEX: F / INCIDENT -> START OF TRAINING (MONTHS): 14 / ICD-10: I63.33

DATE: 10.02.2020 | 12.03.2020 | 08.07.2020 | 28.08.2020

DAYS / # TRAININGS: 31 / 41 | 118 / 82 | 51 / 74

SUBJ. (PCTL): 80.9 | 58.6 | 58.4 | 64.4

OS

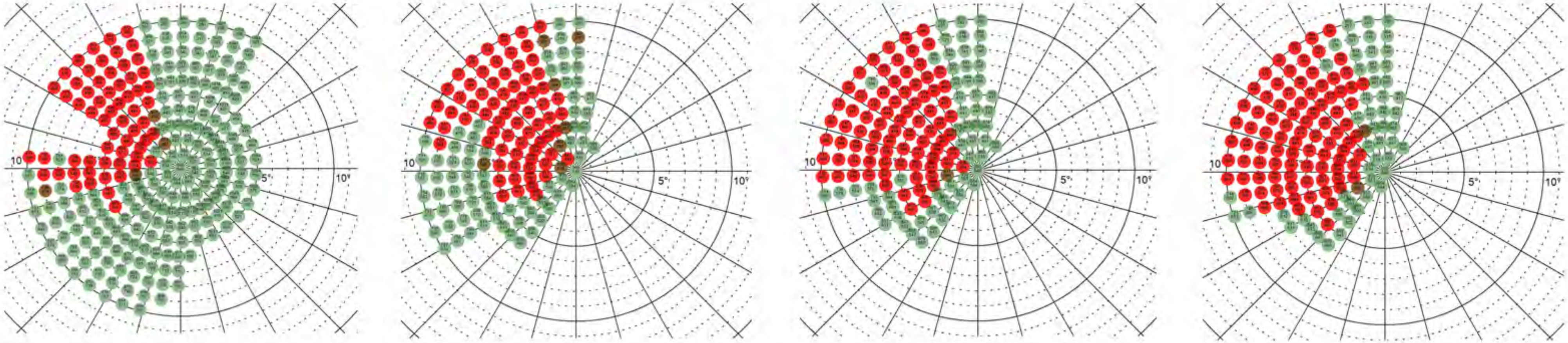

DET. RATE (%): 50.0 | 48.0 | 47.2 | 40.5

OD

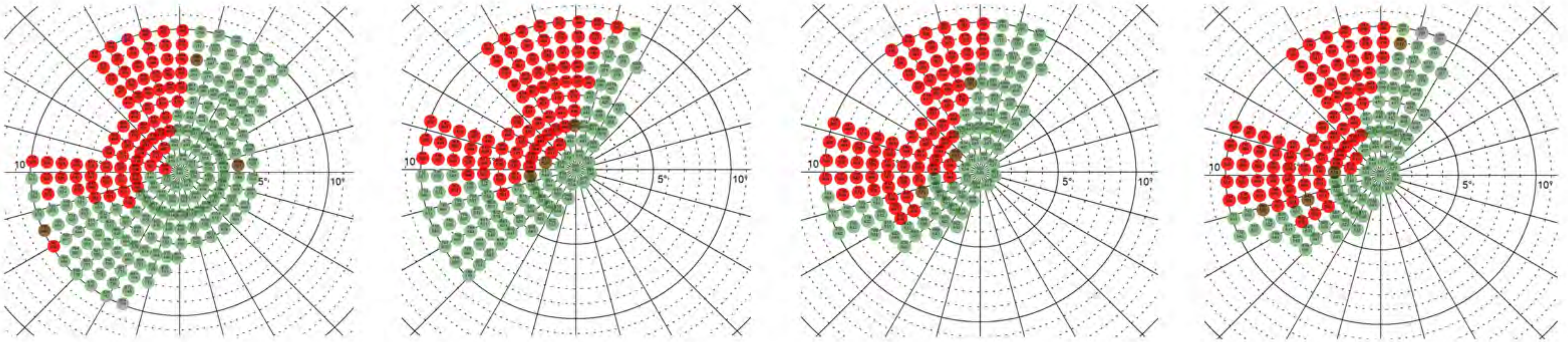

DET. RATE (%): 47.6 | 50.7 | 52.7 | 50.3

DIAGNOSIS WITH “EYE TRACKING BASED VISUAL FIELD ANALYSIS“ (EFA) / DOI: 10.1136/bmjophth-2019-000429

TRAINING WITH “SALZBURG VISUAL FIELD TRAINER“ (SVFT) / DOI: 10.1371/journal.pone.0249762

CODE: 02 / AGE: 76 / SEX: M / INCIDENT -> START OF TRAINING (MONTHS): 202 / ICD-10: I63.33

DATE:	07.02.2020	09.03.2020	22.06.2020
# DAYS / # TRAININGS:	31 / 50	105 / 164	
SUBJ. (PCTL):	48.7	55.5	70.2

OS

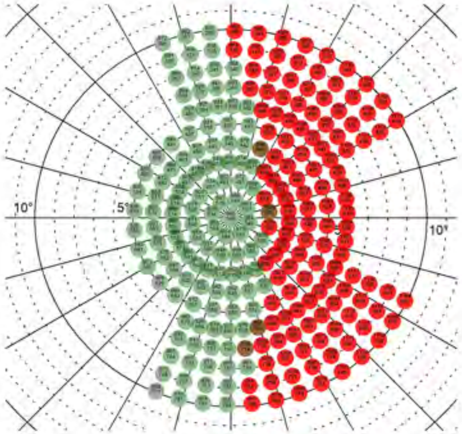
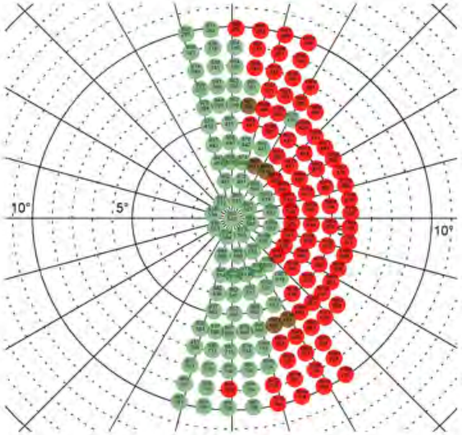
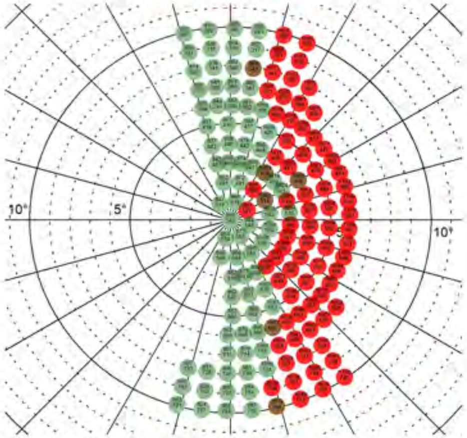

DET. RATE (%):	39.4	47.4	51.6

OD

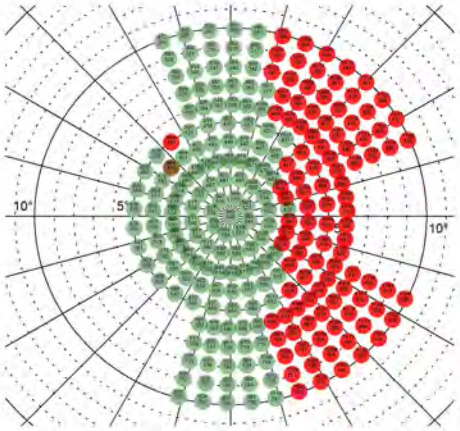
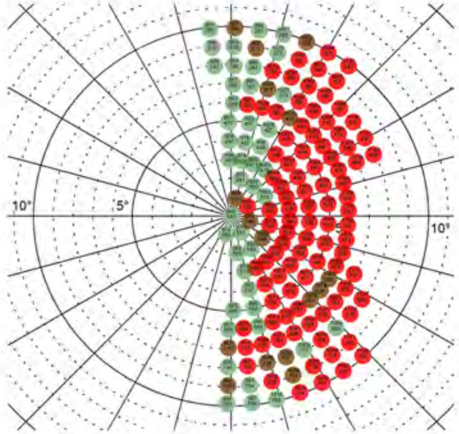
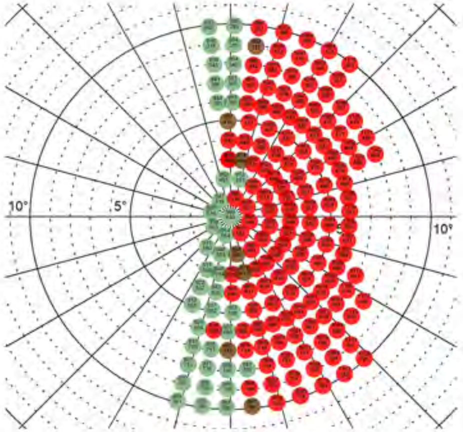

DET. RATE (%):	44.2	33.8	13.3

DIAGNOSIS WITH “EYE TRACKING BASED VISUAL FIELD ANALYSIS“ (EFA) / DOI: 10.1136/bmjophth-2019-000429
TRAINING WITH “SALZBURG VISUAL FIELD TRAINER“ (SVFT) / DOI: 10.1371/journal.pone.0249762

CODE: 03 / AGE: 78 / SEX: F / INCIDENT -> START OF TRAINING (MONTHS): 15 / ICD-10: I63.33

DATE:	05.02.2020		11.03.2020		18.06.2020		07.07.2020
# DAYS / # TRAININGS:		35 / 57		99 / 175		19 / 34	
SUBJ. (PCTL):	59.1		34.3		46.8		43.2

OS

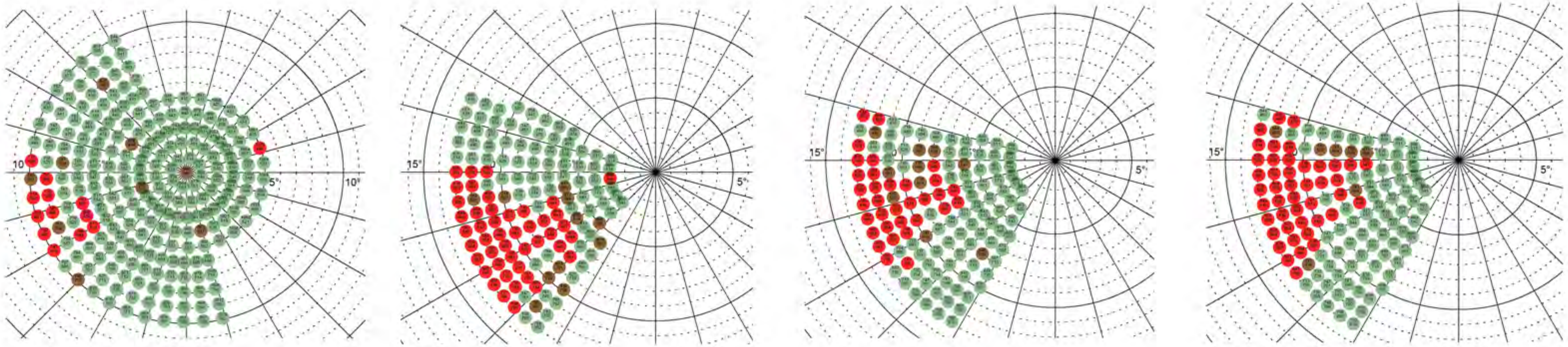

DET. RATE (%):	78.7	71.3	80.1	77.2

OD

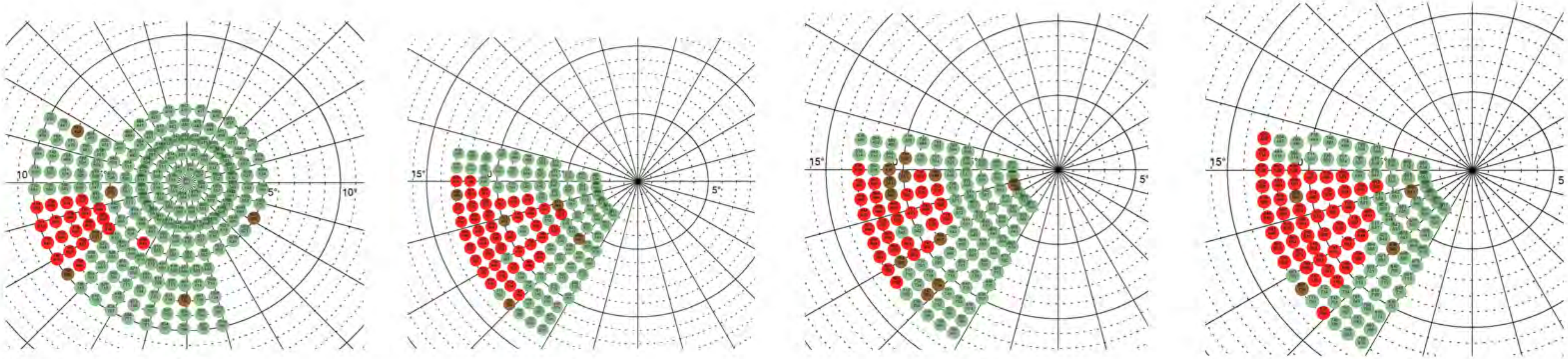

DET. RATE (%):	70.5	77.4	83.6	71.2

DIAGNOSIS WITH “EYE TRACKING BASED VISUAL FIELD ANALYSIS“ (EFA) / DOI: 10.1136/bmjophth-2019-000429

TRAINING WITH “SALZBURG VISUAL FIELD TRAINER“ (SVFT) / DOI: 10.1371/journal.pone.0249762

CODE: 04 / AGE: 64 / SEX: M / INCIDENT -> START OF TRAINING (MONTHS): 39 / ICD-10: I63.33

DATE: 04.02.2020 10.03.2020 19.06.2020

DAYS / # TRAININGS: 35 / 49 101 / 173

SUBJ. (PCTL): 64.2 67.8 62.8

OS

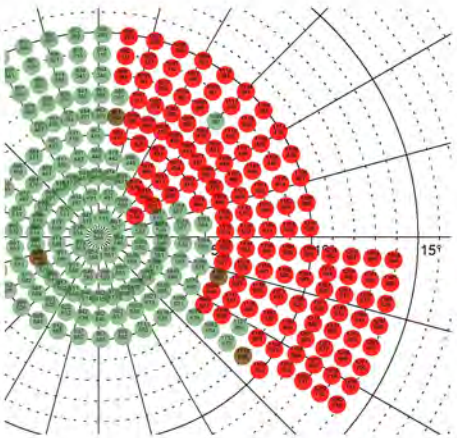

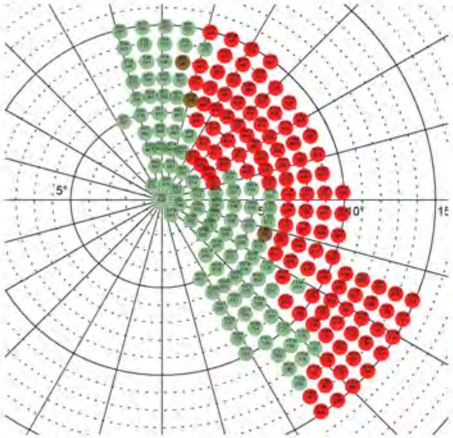

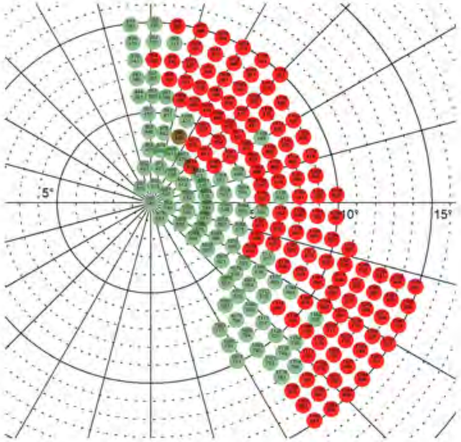

DET. RATE (%): 34.3 39.9 39.9

OD

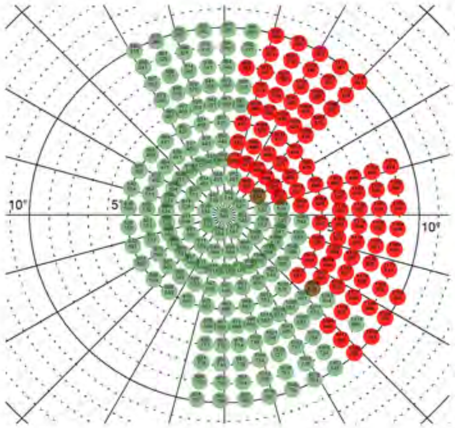

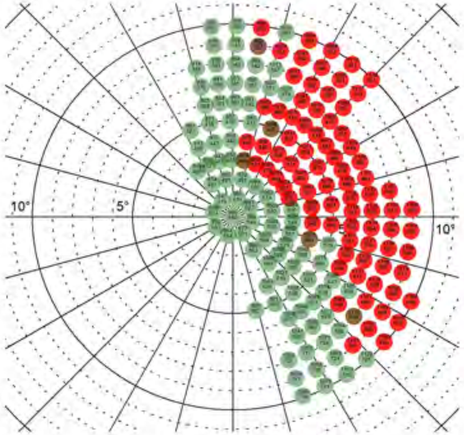

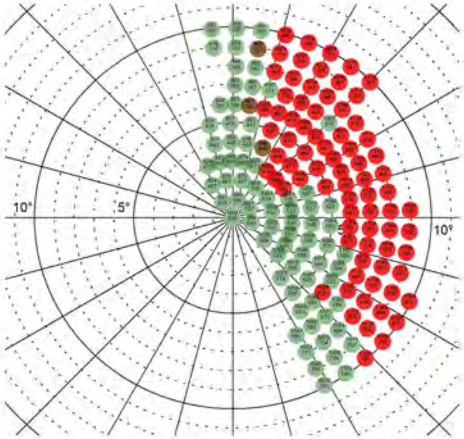

DET. RATE (%): 38.5 46.5 52.1

DIAGNOSIS WITH “EYE TRACKING BASED VISUAL FIELD ANALYSIS“ (EFA) / DOI: 10.1136/bmjophth-2019-000429

TRAINING WITH “SALZBURG VISUAL FIELD TRAINER“ (SVFT) / DOI: 10.1371/journal.pone.0249762

CODE: 05 / AGE: 88 / SEX: M / INCIDENT -> START OF TRAINING (MONTHS): 36 / ICD-10: I63.33

DATE:	28.01.2020	04.03.2020	29.06.2020
# DAYS / # TRAININGS:		36 / 64	117 / 192
SUBJ. (PCTL):	45.9	33.3	64.1

OS

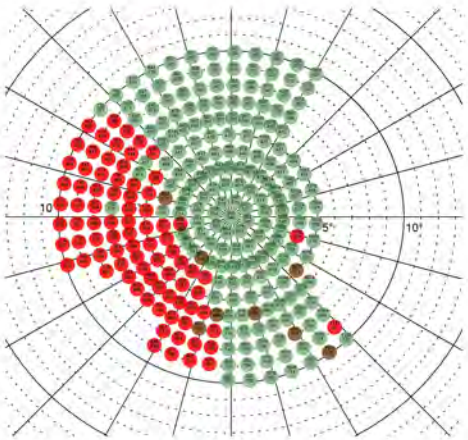

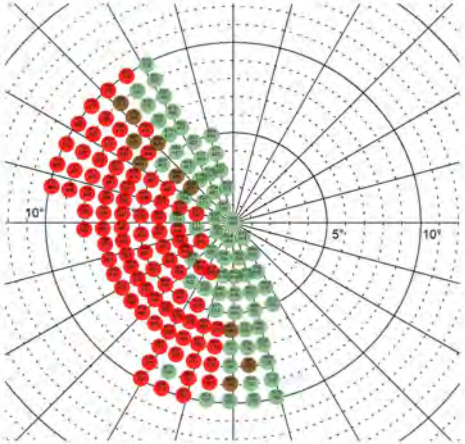

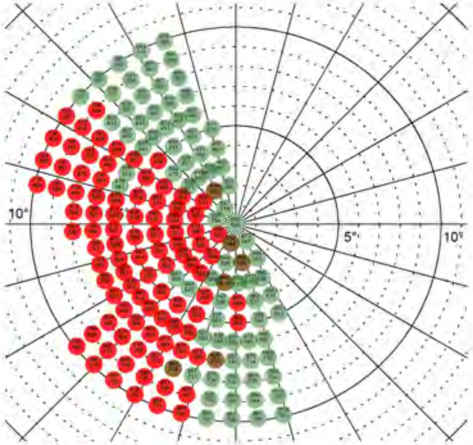

DET. RATE (%):	53.5	43.0	46.8

DIAGNOSIS WITH “EYE TRACKING BASED VISUAL FIELD ANALYSIS“ (EFA) / DOI: 10.1136/bmjophth-2019-000429
TRAINING WITH “SALZBURG VISUAL FIELD TRAINER“ (SVFT) / DOI: 10.1371/journal.pone.0249762

CODE: 06 / AGE: 78 / SEX: M / INCIDENT -> START OF TRAINING (MONTHS): 6 / ICD-10: I63.33

DATE:	12.02.2020	24.06.2020	10.07.2020
# DAYS / # TRAININGS:	133 / 212	16 / 24	
SUBJ. (PCTL):	38.5	46.1	39.4

OS

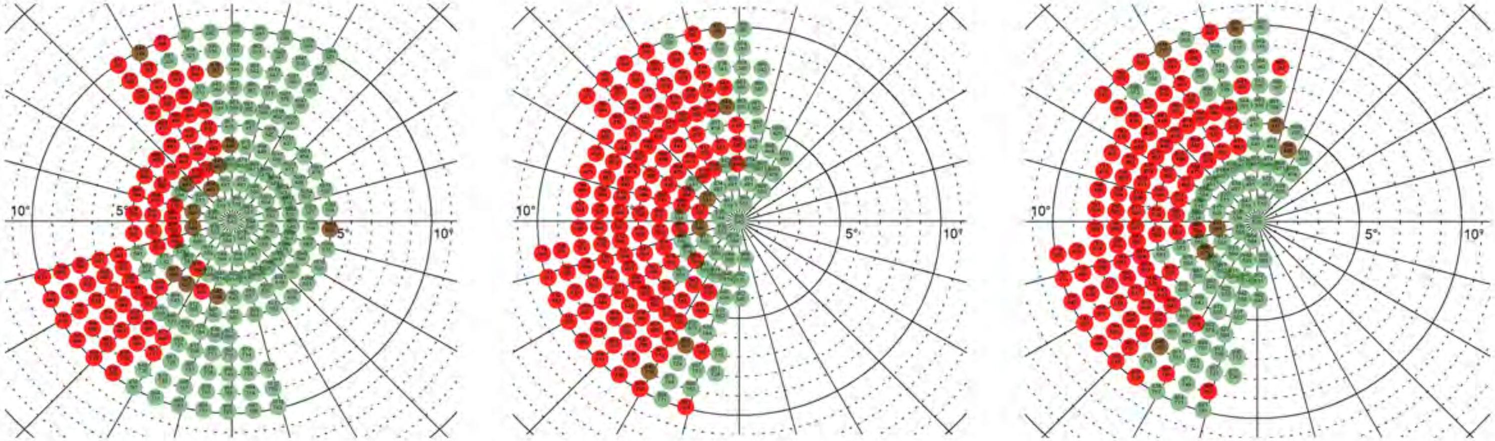

DET. RATE (%):	49.0	38.8	57.6

OD

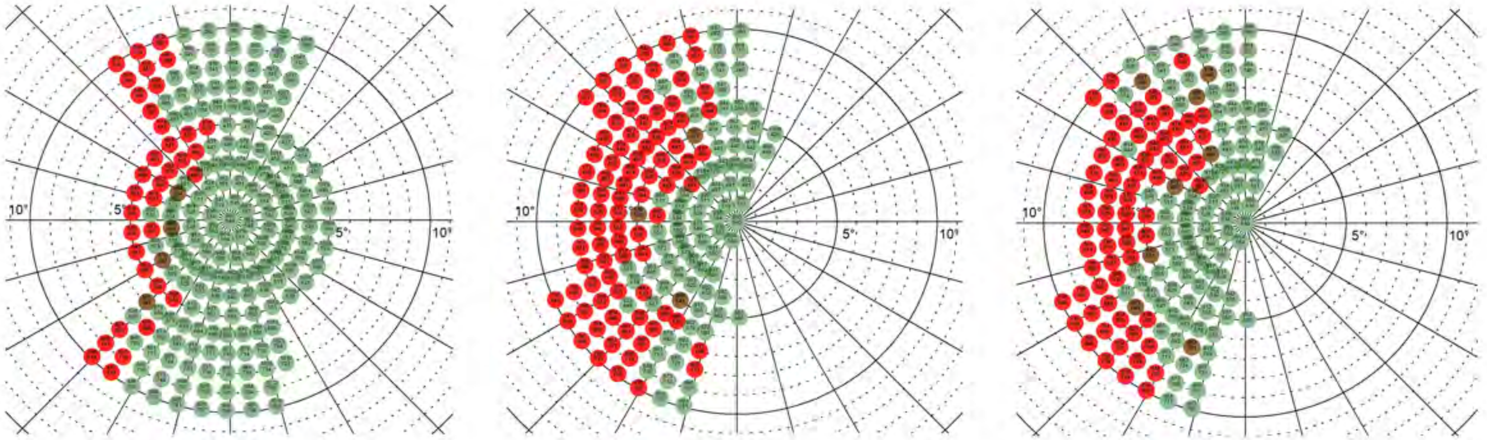

DET. RATE (%):	68.9	66.0	76.9

DIAGNOSIS WITH “EYE TRACKING BASED VISUAL FIELD ANALYSIS“ (EFA) / DOI: 10.1136/bmjophth-2019-000429

TRAINING WITH “SALZBURG VISUAL FIELD TRAINER“ (SVFT) / DOI: 10.1371/journal.pone.0249762

CODE: 08 / AGE: 54 / SEX: M / INCIDENT -> START OF TRAINING (MONTHS): 44 / ICD-10: I63.33

DATE:	31.07.2020	09.10.2020	30.07.2021
# DAYS / # TRAININGS:	70 / 120	294 / 224	
SUBJ. (PCTL):	33.0	39.7	60.3

OS

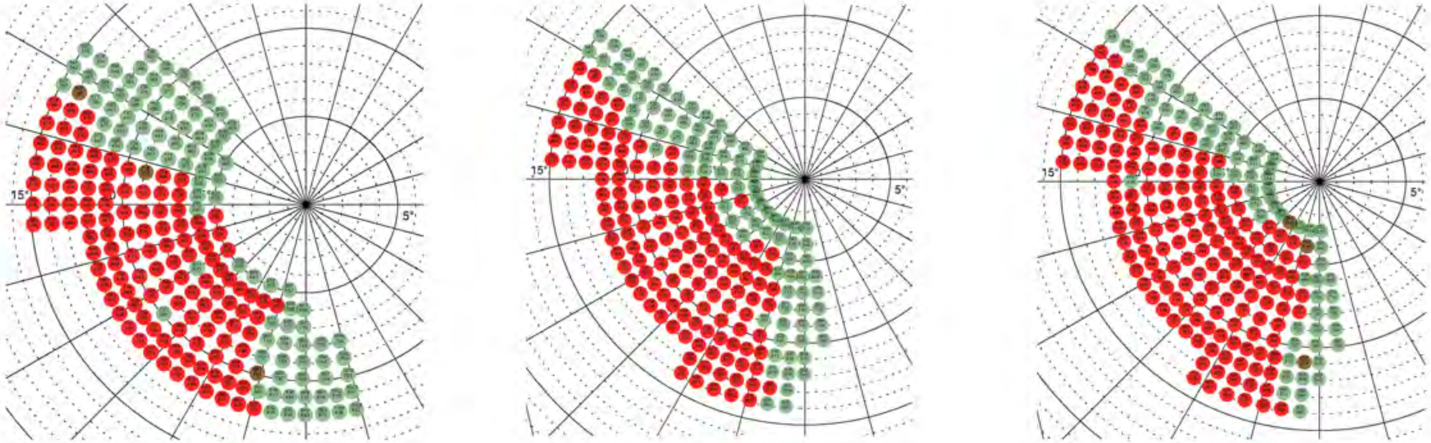

DET. RATE (%): 37.6 — 34.1 — 24.1

OD

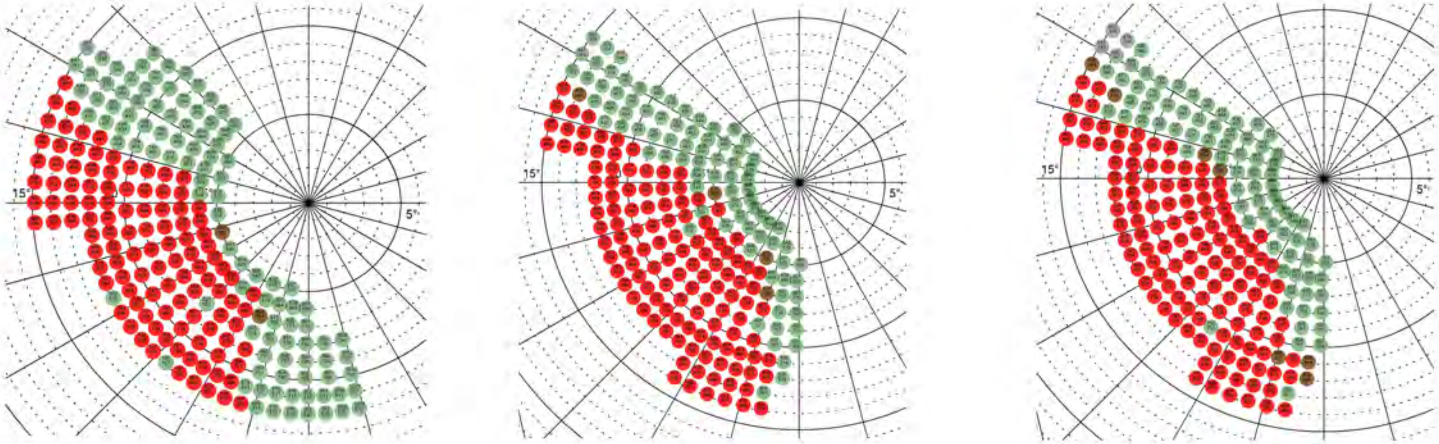

DET. RATE (%): 40.6 — 36.6 — 35.1

DIAGNOSIS WITH "EYE TRACKING BASED VISUAL FIELD ANALYSIS" (EFA) / DOI: 10.1136/bmjophth-2019-000429

TRAINING WITH "SALZBURG VISUAL FIELD TRAINER" (SVFT) / DOI: 10.1371/journal.pone.0249762

CODE: 10 / AGE: 36 / SEX: M / INCIDENT -> START OF TRAINING (MONTHS): 3 / ICD-10: I63.33

DATE:	11.09.2020		03.11.2020	13.01.2021
# DAYS / # TRAININGS:		53 / 102	71 / 124	
SUBJ. (PCTL):	80.6		73.1	74.8

OS

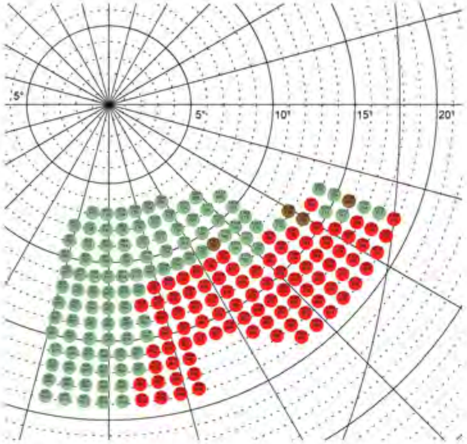
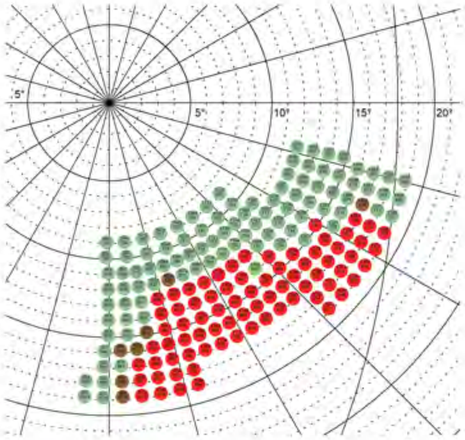
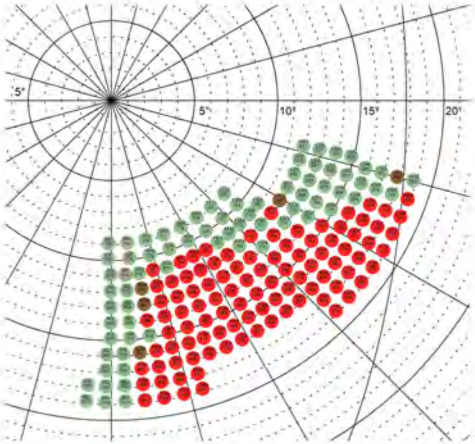

DET. RATE (%):	43.5	51.0	38.0

OD

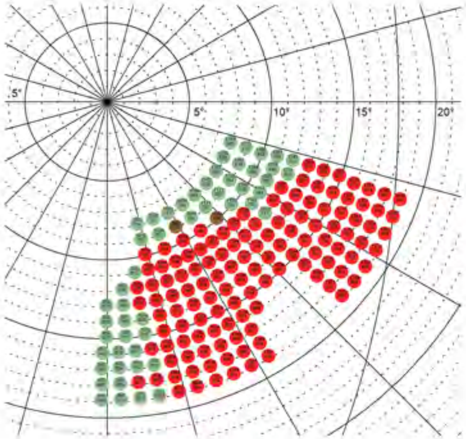
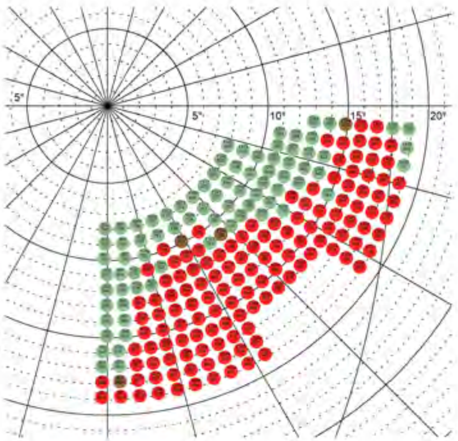
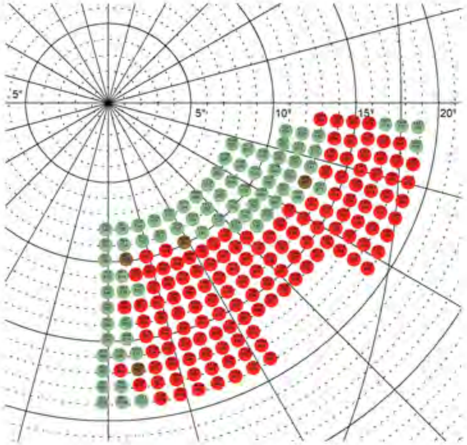

DET. RATE (%):	30.8	34.0	33.4

DIAGNOSIS WITH “EYE TRACKING BASED VISUAL FIELD ANALYSIS“ (EFA) / DOI: 10.1136/bmjophth-2019-000429

TRAINING WITH “SALZBURG VISUAL FIELD TRAINER“ (SVFT) / DOI: 10.1371/journal.pone.0249762

CODE: 11 / AGE: 31 / SEX: M / INCIDENT -> START OF TRAINING (MONTHS): 22 / ICD-10: I63.33

DATE:	13.10.2020		18.12.2020		19.02.2021		16.04.2021
# DAYS / # TRAININGS:		66 / 116		63 / 108		56 / 95	
SUBJ. (PCTL):	50.0		74.9		75.3		85.9

OS

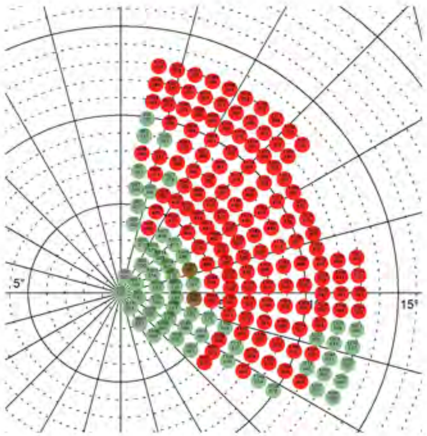
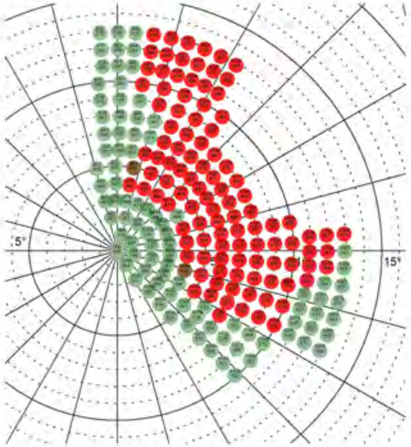
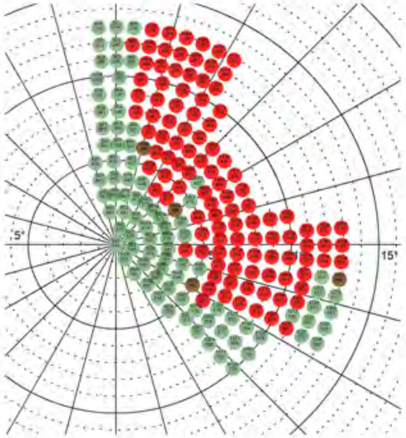
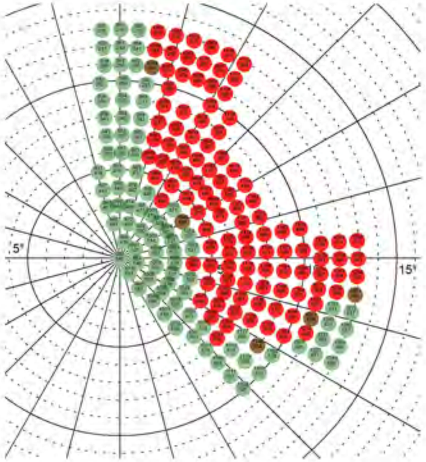

DET. RATE (%): 36.4 | 33.3 | 33.3 | 33.0

OD

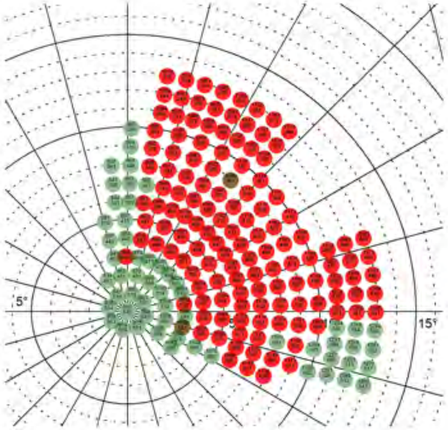
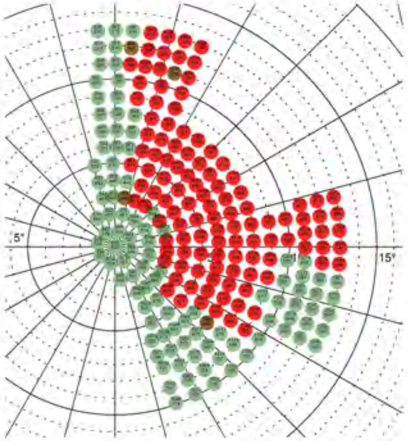
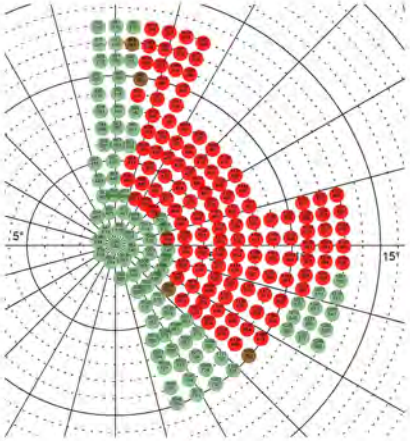
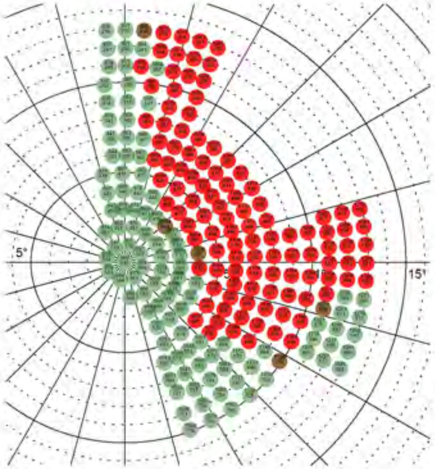

DET. RATE (%): 35.5 | 33.3 | 29.3 | 36.1

DIAGNOSIS WITH “EYE TRACKING BASED VISUAL FIELD ANALYSIS“ (EFA) / DOI: 10.1136/bmjophth-2019-000429

TRAINING WITH “SALZBURG VISUAL FIELD TRAINER“ (SVFT) / DOI: 10.1371/journal.pone.0249762

CODE: 12 / AGE: 54 / SEX: M / INCIDENT -> START OF TRAINING (MONTHS): 13 / ICD-10: I63.33

DATE: 09.03.2021 | 31.05.2021 | 23.08.2021 | 13.10.2021

DAYS / # TRAININGS: 83 / 66 | 84 / 10 | 51 / 6

SUBJ. (PCTL): 18.4 | 20.4 | 47.0 | 73.9

OS

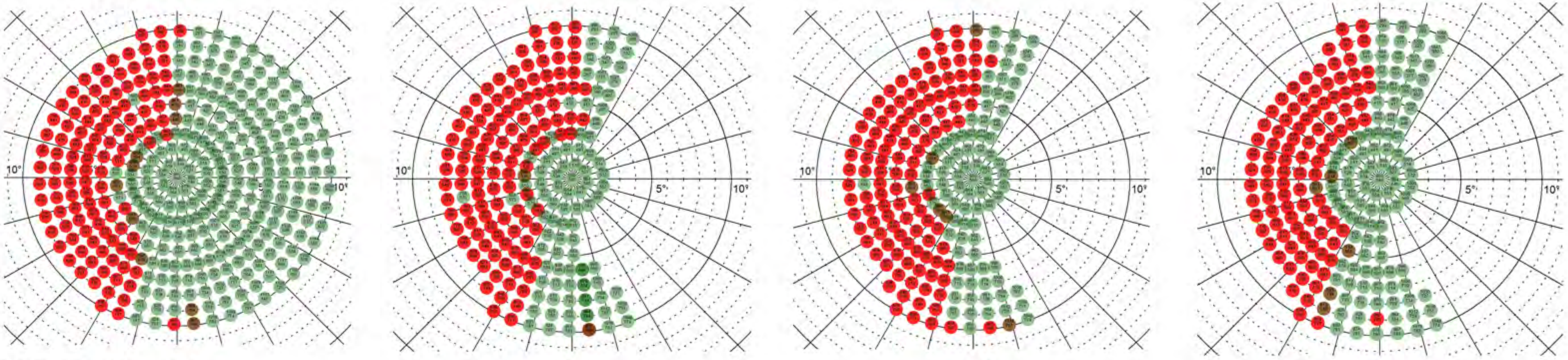

DET. RATE (%): 49.8 | 43.8 | 42.9 | 51.7

OD

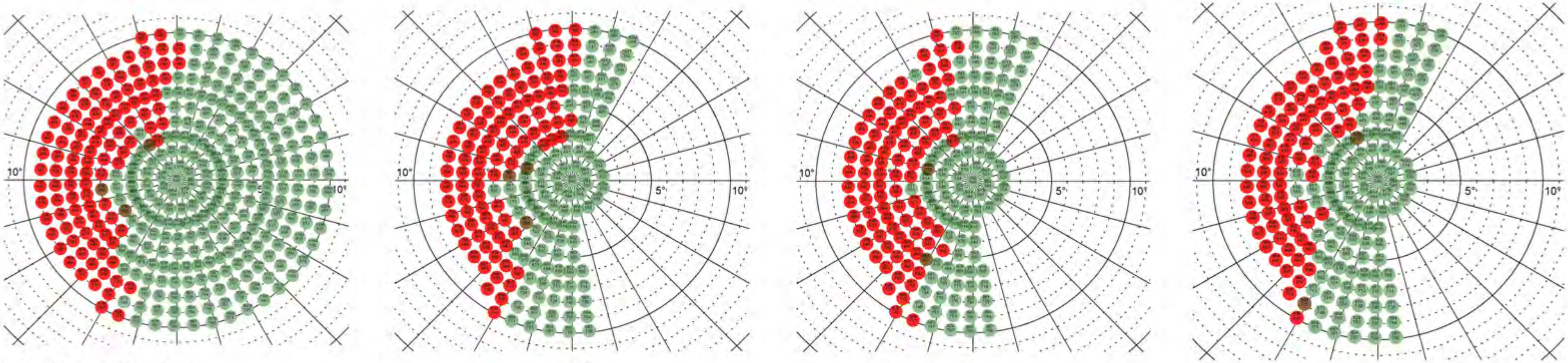

DET. RATE (%): 55.1 | 52.1 | 53.8 | 57.9

DIAGNOSIS WITH “EYE TRACKING BASED VISUAL FIELD ANALYSIS“ (EFA) / DOI: 10.1136/bmjophth-2019-000429

TRAINING WITH “SALZBURG VISUAL FIELD TRAINER“ (SVFT) / DOI: 10.1371/journal.pone.0249762

CODE: 13 / AGE: 53 / SEX: M / INCIDENT -> START OF TRAINING (MONTHS): 15 / ICD-10: I63.33

DATE: 19.03.2021 | 21.05.2021 | 16.07.2021

DAYS / # TRAININGS: 63 / 88 | 56 / 66

SUBJ. (PCTL): 77.1 | 84.3 | 89.4

OS

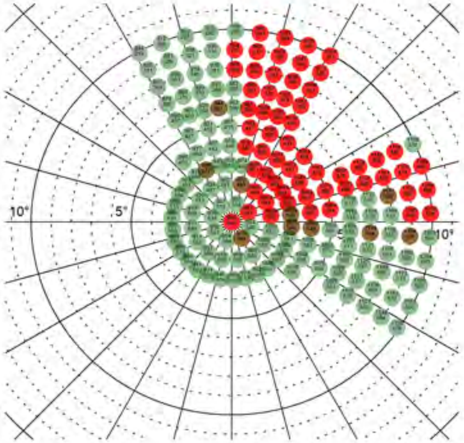

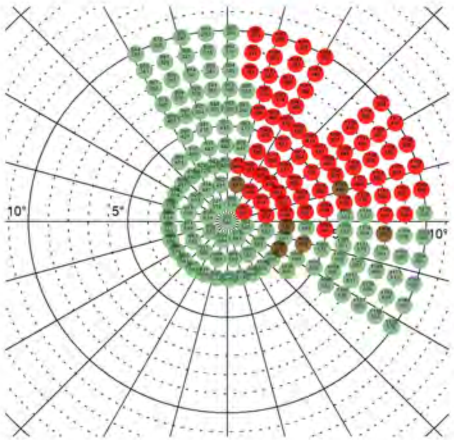

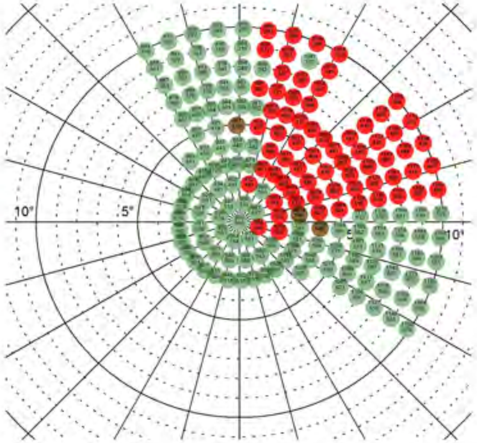

DET. RATE (%): 63.0 | 64.7 | 67.3

OD

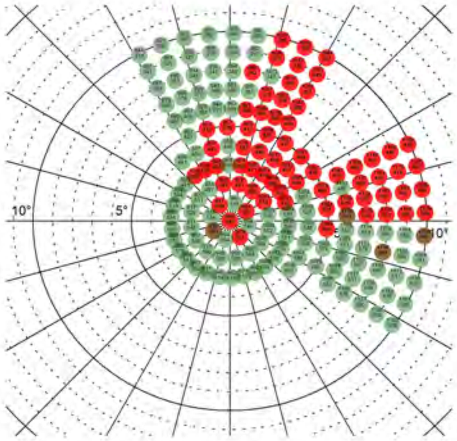

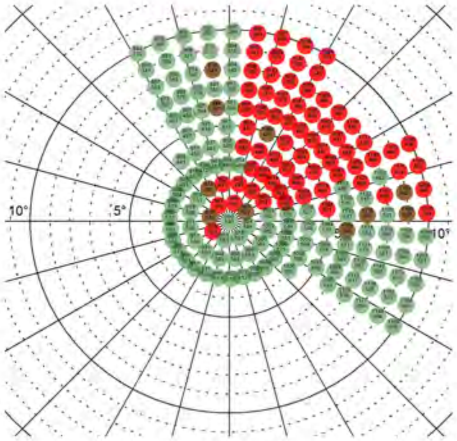

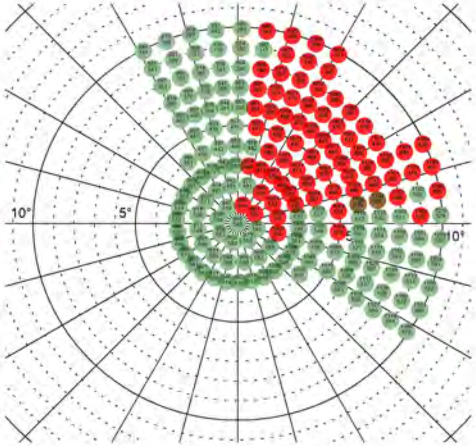

DET. RATE (%): 60.1 | 65.3 | 66.5

DIAGNOSIS WITH “EYE TRACKING BASED VISUAL FIELD ANALYSIS“ (EFA) / DOI: 10.1136/bmjophth-2019-000429

TRAINING WITH “SALZBURG VISUAL FIELD TRAINER“ (SVFT) / DOI: 10.1371/journal.pone.0249762

CODE: 14 / AGE: 53 / SEX: M / INCIDENT -> START OF TRAINING (MONTHS): 15 / ICD-10: I61.3

DATE: 06.04.2021 01.06.2021

DAYS / # TRAININGS: 56 / 84

SUBJ. (PCTL): 14.0 33.4

OS

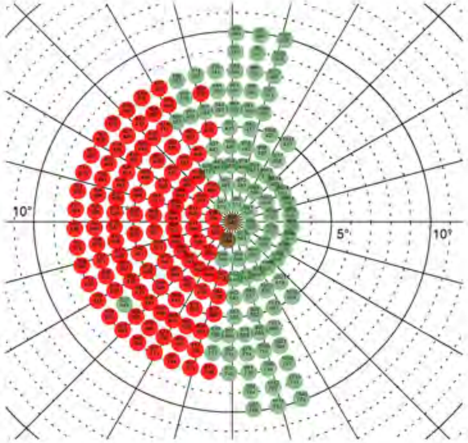

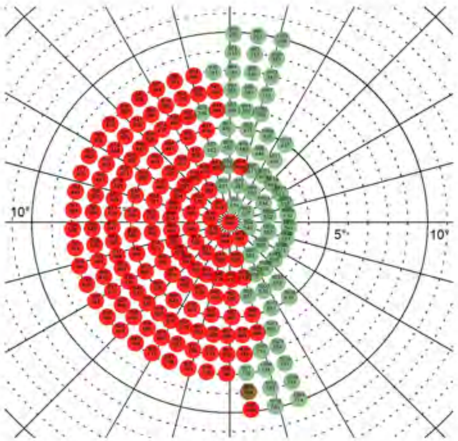

DET. RATE (%): 49.0 32.6

OD

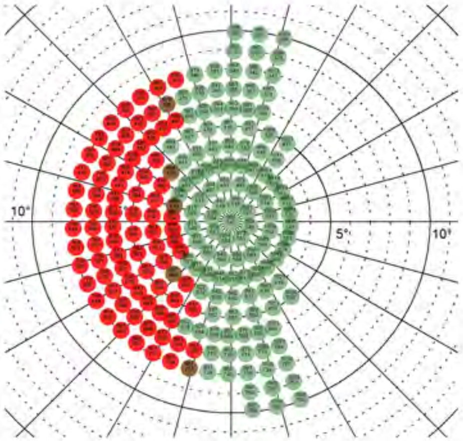

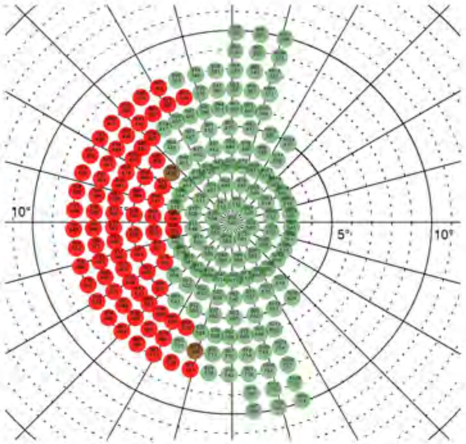

DET. RATE (%): 60.5 64.2

DIAGNOSIS WITH “EYE TRACKING BASED VISUAL FIELD ANALYSIS“ (EFA) / DOI: 10.1136/bmjophth-2019-000429

TRAINING WITH “SALZBURG VISUAL FIELD TRAINER“ (SVFT) / DOI: 10.1371/journal.pone.0249762

CODE: 17 / AGE: 57 / SEX: F / INCIDENT -> START OF TRAINING (MONTHS): 6 / ICD-10: I63.33

DATE:	09.07.2021	30.09.2021	30.11.2021
# DAYS / # TRAININGS:	83 / 132	61 / 91	
SUBJ. (PCTL):	42.0	49.5	48.3

OS

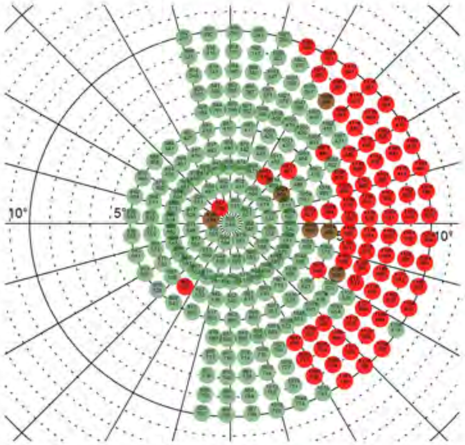

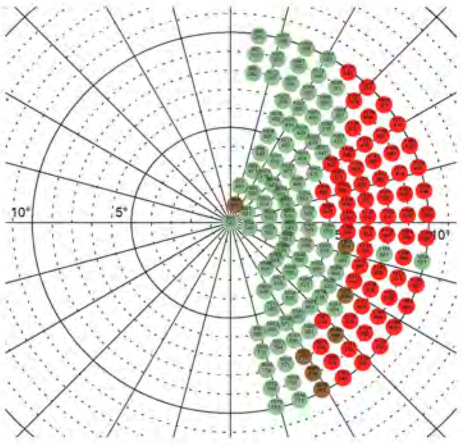

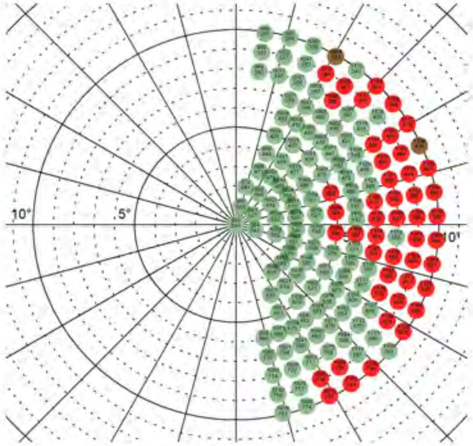

DET. RATE (%):	49.4	59.4	69.7

OD

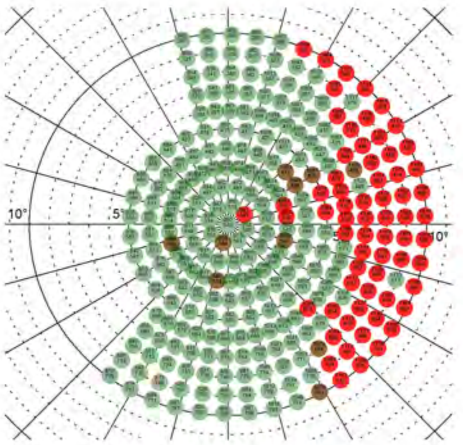

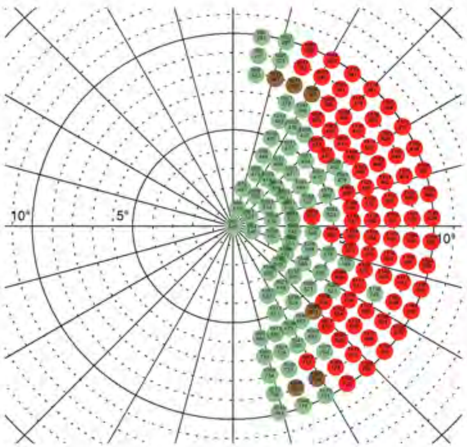

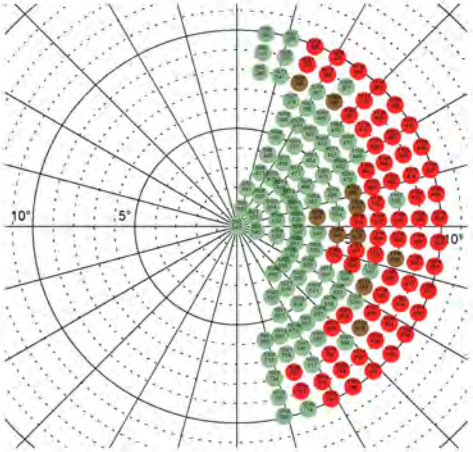

DET. RATE (%):	55.8	48.4	56.8

DIAGNOSIS WITH “EYE TRACKING BASED VISUAL FIELD ANALYSIS“ (EFA) / DOI: 10.1136/bmjophth-2019-000429

TRAINING WITH “SALZBURG VISUAL FIELD TRAINER“ (SVFT) / DOI: 10.1371/journal.pone.0249762

CODE: 19 / AGE: 57 / SEX: M / INCIDENT -> START OF TRAINING (MONTHS): 19 / ICD-10: I61.33, I63.51

DATE: 07.07.2021 21.09.2021

DAYS / # TRAININGS: 76 / 120

SUBJ. (PCTL): 25.2 31.4

OS

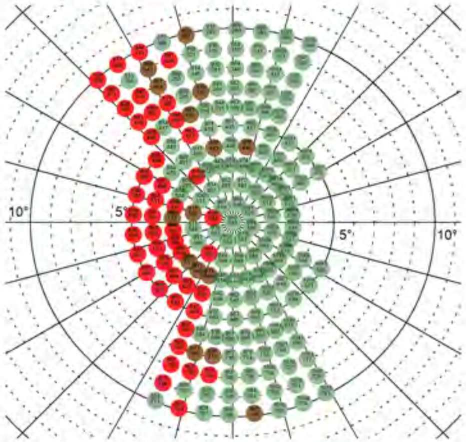

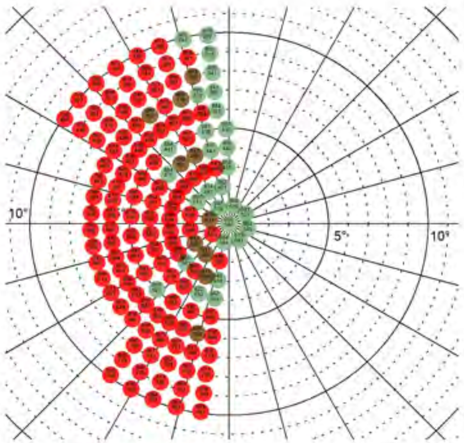

DET. RATE (%): 50.9 31.8

OD

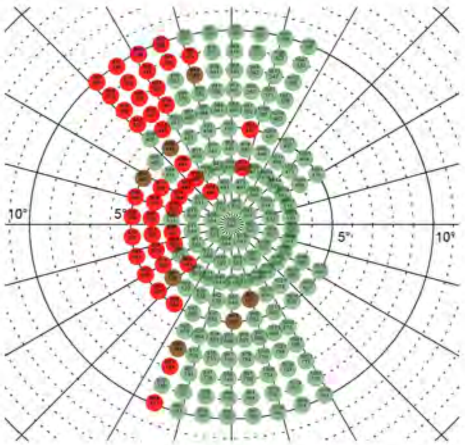

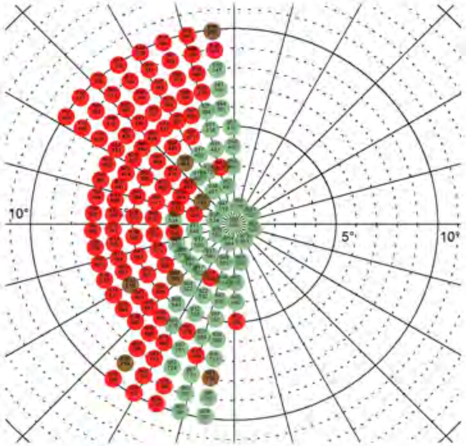

DET. RATE (%): 60.3 47.2

DIAGNOSIS WITH “EYE TRACKING BASED VISUAL FIELD ANALYSIS“ (EFA) / DOI: 10.1136/bmjophth-2019-000429

TRAINING WITH “SALZBURG VISUAL FIELD TRAINER“ (SVFT) / DOI: 10.1371/journal.pone.0249762

CODE: 20 / AGE: 39 / SEX: F / INCIDENT -> START OF TRAINING (MONTHS): 3 / ICD-10: I63.33

DATE:	29.07.2021	29.09.2021	29.11.2021
# DAYS / # TRAININGS:	62 / 98	61 / 67	
SUBJ. (PCTL):	78.4	69.9	75.2

OS

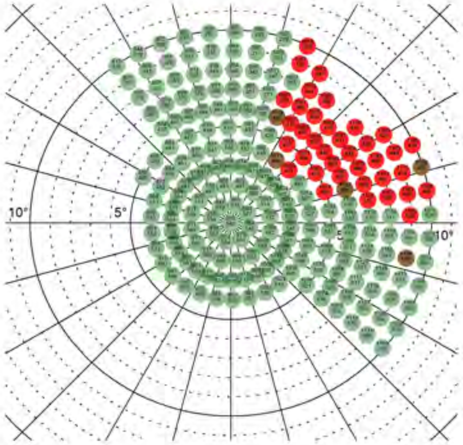
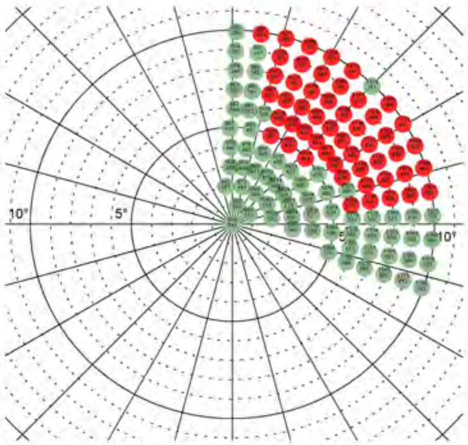
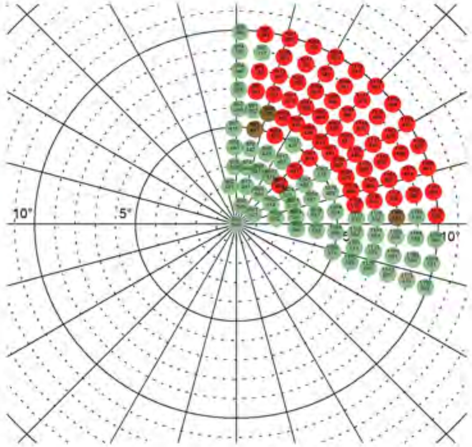

DET. RATE (%):	63.1	59.8	53.7

OD

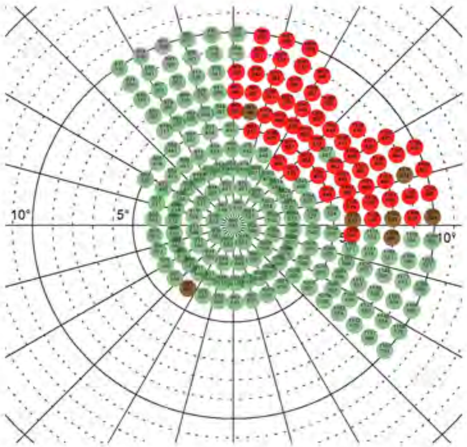
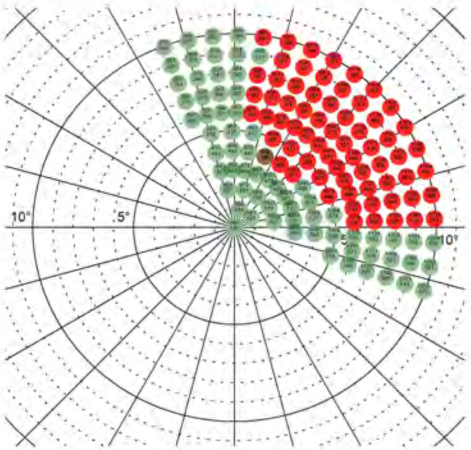
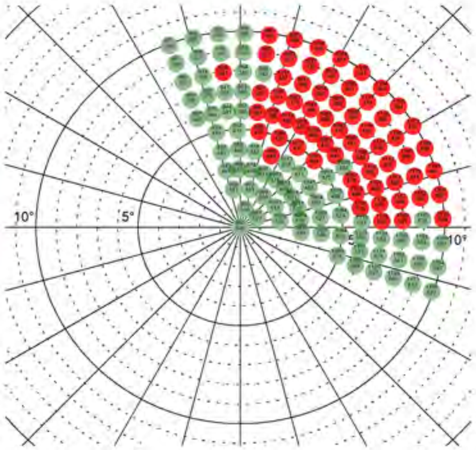

DET. RATE (%):	52.0	54.0	57.6

DIAGNOSIS WITH “EYE TRACKING BASED VISUAL FIELD ANALYSIS“ (EFA) / DOI: 10.1136/bmjophth-2019-000429
TRAINING WITH “SALZBURG VISUAL FIELD TRAINER“ (SVFT) / DOI: 10.1371/journal.pone.0249762

CODE: 22 / AGE: 35 / SEX: M / INCIDENT -> START OF TRAINING (MONTHS): 4 / ICD-10: I63.33

DATE:	04.10.2021		07.12.2021		08.02.2022
# DAYS / # TRAININGS:		64 / 93		63 / 35	
SUBJ. (PCTL):	59.7		29.6		82.4

OS

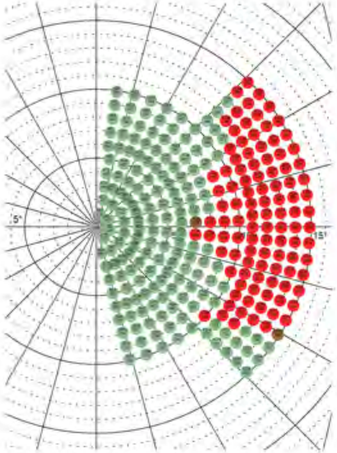

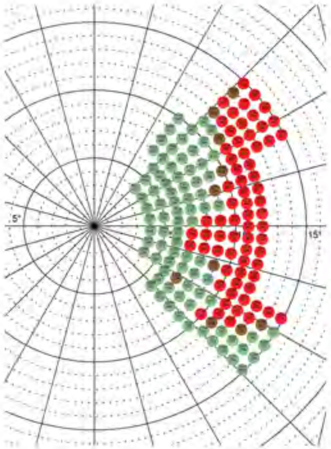

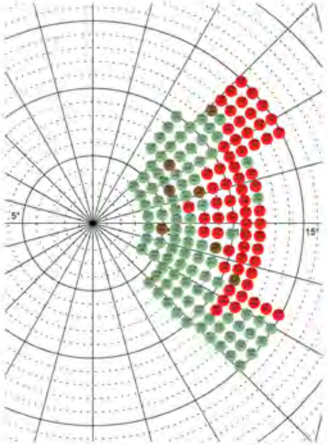

DET. RATE (%): 55.6 | 54.4 | 59.7

OD

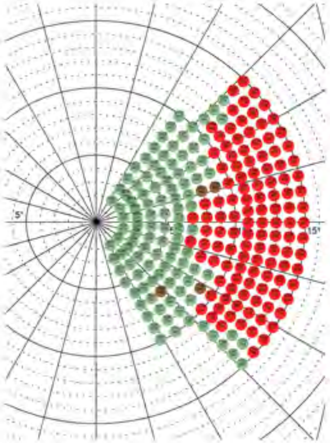

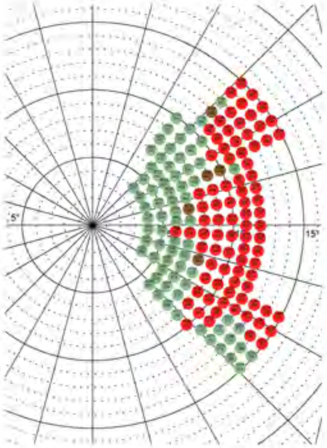

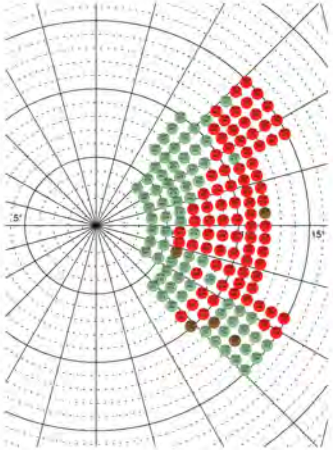

DET. RATE (%): 46.3 | 45.9 | 47.2

DIAGNOSIS WITH “EYE TRACKING BASED VISUAL FIELD ANALYSIS“ (EFA) / DOI: 10.1136/bmjophth-2019-000429

TRAINING WITH “SALZBURG VISUAL FIELD TRAINER“ (SVFT) / DOI: 10.1371/journal.pone.0249762
